# Interferon induced circRNAs escape herpesvirus host shutoff and suppress lytic infection

**DOI:** 10.1038/s44319-023-00051-z

**Published:** 2024-01-23

**Authors:** Sarah E Dremel, Takanobu Tagawa, Vishal N Koparde, Carmen Hernandez-Perez, Jesse H Arbuckle, Thomas M Kristie, Laurie T Krug, Joseph M Ziegelbauer

**Affiliations:** 1 HIV and AIDS Malignancy Branch, National Cancer Institute, Bethesda, 20892 USA; 2 CCR Collaborative Bioinformatics Resource, National Cancer Institute, Bethesda, 20892 USA; 3 Frederick National Laboratory for Cancer Research Advanced Biomedical Computational Sciences, Leidos Biomedical Research, Inc., Frederick, 21701 USA; 4 Laboratory of Viral Diseases, National Institute of Allergy and Infectious Diseases, Bethesda, 20892 USA

**Keywords:** Circular RNAs; Herpesviruses; Host Shutoff; Interferon-stimulated Genes, Immunology, Microbiology, Virology & Host Pathogen Interaction, RNA Biology

## Abstract

To globally profile circRNAs, we employ RNA-Sequencing paired with chimeric junction analysis for alpha-, beta-, and gamma-herpesvirus infection. We find circRNAs are, as a population, resistant to host shutoff. We validate this observation using ectopic expression assays of human and murine herpesvirus endoribonucleases. During lytic infection, four circRNAs are commonly induced across all subfamilies of human herpesviruses, suggesting a shared mechanism of regulation. We test one such mechanism, namely how interferon-stimulation influences circRNA expression. 67 circRNAs are upregulated by either interferon-β or -γ treatment, with half of these also upregulated during lytic infection. Using gain and loss of function studies we find an interferon-stimulated circRNA, circRELL1, inhibits lytic Herpes Simplex Virus-1 infection. We previously reported circRELL1 inhibits lytic Kaposi sarcoma-associated herpesvirus infection, suggesting a pan-herpesvirus antiviral activity. We propose a two-pronged model in which interferon-stimulated genes may encode both mRNA and circRNA with antiviral activity. This is critical in cases of host shutoff, such as alpha- and gamma-herpesvirus infection, where the mRNA products are degraded but circRNAs escape.

## Synopsis

Comparative profiling of circRNAs during alpha-, beta-, and gamma-herpesvirus infection establishes circRNAs as host shutoff escapees. circRELL1 inhibits herpesvirus replication and is induced by infection and interferon-stimulation.

- Levels of circRNAs are globally profiled during alpha-, beta-, and gamma-herpesvirus infection.
- circRNAs are resistant to herpesvirus endoribonucleases.
- 67 circRNAs are upregulated by either interferon-β or -γ treatment.
- Interferon-stimulated circRELL1 inhibits HSV-1 lytic infection.

## Abstract (web summary)

Comparative profiling of circRNAs during alpha-, beta-, and gamma-herpesvirus infection establishes circRNAs as host shutoff escapees. circRELL1 inhibits herpesvirus replication and is induced by infection and interferon-stimulation.

## Graphical abstract

**Figure:**
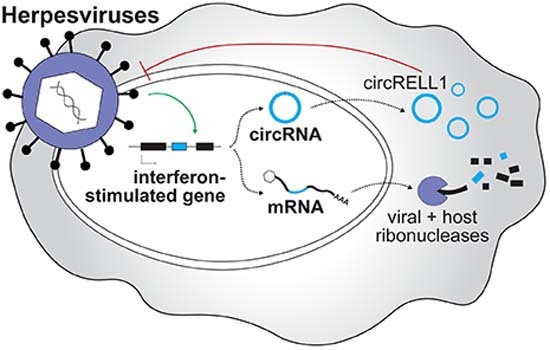

**Figure:**
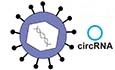

## Introduction

Herpesviridae is a family of large, double-stranded DNA viruses with a biphasic life cycle, a lytic (replicative) and latent (quiescent, immune evasive) phase. There are nine species known to infect humans, including the alpha-herpesvirus Herpes Simplex Virus-1 (HSV-1), beta-herpesvirus human cytomegalovirus (HCMV), and gamma-herpesvirus Kaposi sarcoma-associated herpesvirus (KSHV). Herpesviruses are a major public health concern with individuals testing seropositive for at least three of the nine species by adulthood (Ablashi et al, 1995; Baillargeon et al, 2000; Bate et al, 2010; Bradley et al, 2013; Dowd et al, 2013; Zhang et al, 2022). Infection is asymptomatic for many individuals but, in cases of immune-compromise—such as transplant recipients, neonates, and those with HIV/AIDS—these viruses have devastating effects. HSV-1 commonly causes recurrent oral and genital lesions, but can also cause herpes keratitis, herpetic whitlow, and encephalitis (Gopinath et al, 2023). HCMV is the most common congenital infection in addition to a severe opportunistic infection in transplant recipients and individuals with HIV/AIDS (Griffiths and Reeves, 2021). KSHV is the etiological agents of several cancers including Kaposi sarcoma and primary effusion lymphoma (Cesarman et al, 2019). Murine gamma-herpesvirus 68 (MHV68) has close genetic homology to KSHV and serves as a tractable animal model for pathogenesis (Dong et al, 2017; Wang et al, 2021). To date only varicella zoster virus has an FDA-approved vaccine. In addition, we lack antivirals capable of targeting the latent reservoir and there is no therapeutic agent capable of clearing these viruses.

Viruses evolve unique mechanisms to invade hosts, alter cellular pathways, and redirect cellular factors for viral processes. In parallel, the host employs a barrage of proteins and RNA species to combat infection. An emerging class of transcripts, circular RNAs (circRNA), has recently been implicated in this host-pathogen arms race (Chen et al, 2017; Harper et al, 2022; Li et al, 2017; Tagawa et al, 2023; Yao et al, 2021a). CircRNAs are single-stranded RNAs circularized by 5’ to 3′ covalent linkages called back-splice junctions (BSJs). High-throughput sequencing paired with chimeric transcript analysis enables global circRNA detection and quantification (Szabo and Salzman, 2016). These techniques find circRNAs to be ubiquitously expressed in an array of organisms and tissues (Maass et al, 2017; Salzman et al, 2013). CircRNAs are also expressed by viruses, including KSHV, Epstein Barr Virus (EBV), human papillomavirus, Merkel cell polyomavirus, hepatitis B virus, and respiratory syncytial virus (Abere et al, 2020; Tagawa et al, 2018; Toptan et al, 2018; Ungerleider et al, 2018; Yao et al, 2021b; Zhao et al, 2019; Zhou et al, 2018). The mechanism underlying host circRNA synthesis, back-splicing, is catalyzed by the spliceosome and regulated by RNA binding proteins (RBPs) and tandem repeat elements which mediate interaction of BSJ flanking sequences (Ashwal-Fluss et al, 2014; Conn et al, 2015; Jeck et al, 2013; Starke et al, 2015). CircRNAs function as miRNA sponges, protein scaffolds, and transcriptional enhancers (Kristensen et al, 2019). CircRNAs are generally classified as noncoding RNAs (ncRNAs), although they possess the capacity for cap-independent translation (Chen and Sarnow, 1995; Legnini et al, 2017; Pamudurti et al, 2017). Recently, we identified a host circRNA, circRELL1, that increased the growth of KSHV-infected cells while suppressing the lytic cycle, thereby promoting the viral latency program (Tagawa et al, 2023). Additional host circRNAs modulate viral infection (circHIPK3-KSHV, circPSD3-Hepatitis C virus) and are implicated in virus-driven tumorigenesis (circARFGEF1-KSHV, circNBEA-Hepatitis B virus) (Chen et al, 2020; Harper et al, 2022; Huang et al, 2017; Yao et al, 2021a). Furthermore, circRNAs made by spliceosome-independent mechanisms leads to activation of the pattern recognition receptor (PRR), RIG-I (Chen et al, 2017). Another report found that circRNAs, as a class, sequester the RBP encoded by interleukin enhancer-binding factor 3 (NF90/NF110) and this axis modulates vesicular stomatitis virus infection (Li et al, 2017).

As circRNAs lack ends, they are generally resistant to exoribonucleases with approximately 2.5-fold longer half-lives than their linear counterparts (Enuka et al, 2016; Memczak et al, 2013). CircRNAs are also more stable in the extracellular space, a feature which has led to much interest in their potential use as a diagnostic biomarker (Wang and Liu, 2022). Circularity, however, does not prevent susceptibility of circRNAs to endoribonucleases (endoRNases) such as RNase L and RNase P (Liu et al, 2019; Park et al, 2019). Herpesviruses also express endoRNases, e.g., HSV-1 virion host shutoff (vhs), EBV BamHI fragment G leftward open reading frame 5 (BGLF5), KSHV shutoff and exonuclease (SOX), and MHV68 murine SOX (muSOX). These viral proteins drive a phenomenon called “host shutoff”, which, in part, ablates the immune response by degrading interferon-stimulated genes (Abernathy and Glaunsinger, 2015; Smiley, 2004). The viral endoRNases display broad nucleolytic activity in vitro relying on viral and host protein adapters in vivo to fine tune their RNA substrates (Daly et al, 2020). These adapters facilitate a preference for translationally competent RNA leaving ncRNA enriched in the escapee population (Abernathy et al, 2014; Covarrubias et al, 2011; Feng et al, 2005; Gaglia et al, 2012; Page and Read, 2010; Shiflett and Read, 2013). Circularity itself provides some protection from vhs cleavage in vitro, however circRNAs containing an internal ribosome entry site can still be targeted (Shiflett and Read, 2013). In the context of HSV-1 infection, a recent study reported enrichment of circRNAs relative to their colinear gene products, which was not observed in the context of a vhs-null virus (Friedl et al, 2022).

To define host circRNAs commonly regulated by herpesviruses, we performed comparative circRNA expression profiling of cells infected with alpha- (HSV-1), beta- (HCMV), and gamma-herpesviruses (KSHV; MHV68, a murine model of KSHV). We profiled cell culture and animal models, spanning lytic and latent infection. During lytic HSV-1, KSHV, and MHV68 infection, circRNAs were, as a population, unaffected by host shutoff. Ectopic expression assays with human and murine herpesvirus endoRNases confirmed this observation. This agrees with prior reports regarding HSV-1 vhs-mediated decay (Friedl et al, 2022; Shiflett and Read, 2013) and expands the observation to gamma-herpesvirus endoRNases. We identified four human and twelve murine circRNAs commonly upregulated after infection across subfamilies of herpesviruses. The most upregulated pathways in our models of HSV-1, HCMV, and KSHV lytic infection were related to immunity. Thus, we examined if circRNA expression was affected by treatment with various immune stimuli (LPS, poly I:C, CpG) or type I and II interferons (IFN). 67 circRNAs were upregulated by IFN treatment, with half of these also upregulated during viral infection. Finally, we tested if one of these interferon-stimulated circRNAs, circRELL1, echoed the antiviral function of its colinear gene product. Using gain and loss of function studies, circRELL1 was found to inhibit lytic HSV-1 infection. These results echo our prior finding, that circRELL1 inhibits lytic KSHV infection (Tagawa et al, 2023), and hints at a common mechanism of action that spans disparate cell types (fibroblast vs. endothelial) and viruses (alpha vs. gamma-herpesviruses). Our data suggests this class of host shutoff escapees may have largely unprobed potential as immunologic effectors.

## Results

### CircRNA profiling of alpha-, beta-, and gamma-herpesvirus infection

**Figure 1:**
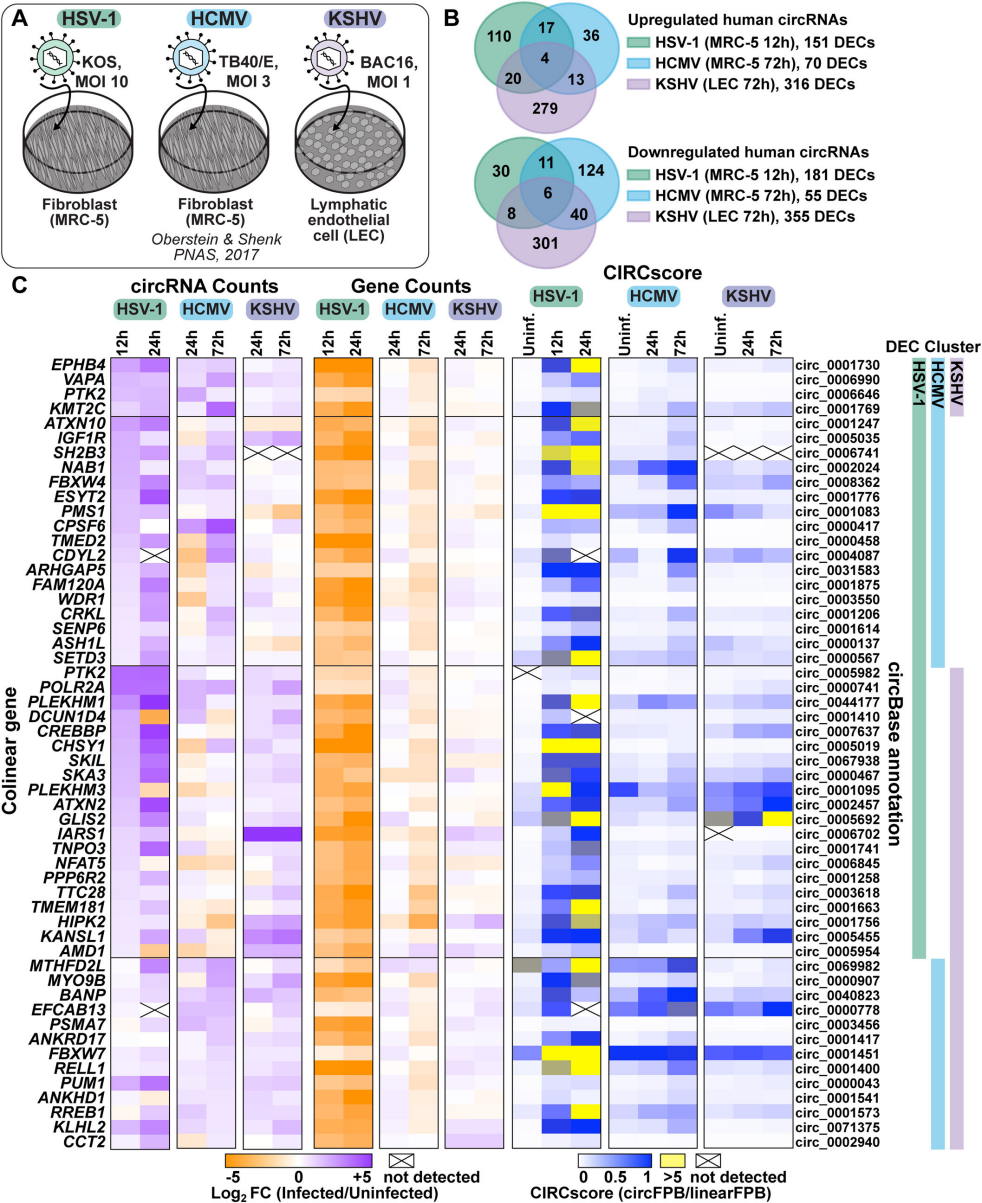
Human circRNAs upregulated in de novo lytic infection models. (**A**) Infographic for infection models used in this study. (**B**) Overlap of differentially expressed circRNAs (DECs) detected by bulk RNA-Seq from HSV-1 (*n* = 2–4), HCMV (*n* = 2), and KSHV (*n* = 2) infection. (**C**) Heatmaps for DECs which overlap between viruses, with DEC clusters indicating which model the circRNA was found to be significantly upregulated within. Data is plotted as circRNA counts (log_2_FC), Gene counts (log_2_FC), or CIRCscore (circFPB (fragments per billion mapped bases)/linearFPB). Data Information: Bulk RNA-Seq data was normalized to ERCC spike-in controls. Log_2_FC is relative to a paired uninfected control. Differential expression *p*-values were calculated using RankProd non-parametric permutation tests. DECs were those with raw back splice junction (BSJ) count across the sample set >10, log_2_FC > 0.5 or <−0.5, and *p*-value < 0.05. Heatmap values are the average of biological replicates.

As alternative splicing products, circRNAs share almost complete sequence identity with their linear counterparts derived from the same gene. We used CIRCExplorer3-CLEAR to quantify the unique sequence of circRNAs, namely the 5′ to 3′ back splice junctions (Ma et al, 2019). CIRCExplorer3 also calculates CIRCscore, the number of reads spanning circRNA BSJs (circ fragments per billion mapped bases, circFPB) against reads spanning mRNA forward splice junctions (linear fragments per billion mapped bases, linearFPB). We profiled RNA-Seq data from alpha-, beta-, and gamma-herpesvirus infection, in cell culture and animal models (Figs. 1A, EV1A and EV2A). The data was a combination of our own RNA-Seq data (HSV-1, KSHV, MHV68) in addition to a previously published dataset (HCMV) (Data ref: Oberstein and Shenk, 2017). For all RNA-Seq, excluding the HCMV dataset, ERCC (External RNA Controls Consortium) spike-ins were used to control for global transcriptomic shifts caused by infection. We have summarized all the circRNA and gene expression profiling as interactive data tables (Dataset EV1–6) with their utility demonstrated in Fig. EV3.

**Figure EV1:**

Murine circRNAs upregulated in HSV-1 infection models. (**A**) Infographic for murine HSV-1 infection models used in this study. (**B**) Overlap of upregulated murine circRNA detected by bulk RNA-Seq from HSV-1 latency (TG, *n* = 4), explant-induced reactivation (TG explant, *n* = 3), and drug-enhanced reactivation (TG explant + JQ1, *n* = 2). (**C**) Heatmaps for upregulated murine circRNAs, plotted as circRNA counts (log_2_FC), Gene counts (log_2_FC), or CIRCScore (circFPB/linearFPB). Data Information: Bulk RNA-Seq data was normalized to ERCC spike-in controls. Log_2_FC is relative to a paired uninfected control. Differential expression *p*-values were calculated using RankProd non-parametric permutation tests. DECs were those with raw back splice junction (BSJ) count across the sample set >10, log_2_FC > 0.5, and *p*-value < 0.05. Heatmap values are the average of biological replicates.

**Figure EV2:**

Murine circRNAs upregulated in MHV68 infection models. (**A**) Infographic for murine MHV68 infection models used in this study. (**B**) Overlap of upregulated murine circRNA detected by bulk RNA-Seq from MHV68 de novo infection (NIH 3T3, *n* = 2) and drug-induced reactivation (HE-RIT, *n* = 2). (**C**) Heatmaps for upregulated murine circRNAs, plotted as circRNA counts (log_2_FC), Gene counts (log_2_FC), or CIRCScore (circFPB/linearFPB). Bulk RNA-Seq data was normalized to ERCC spike-in controls. Log_2_FC is relative a paired uninfected or uninduced control. Differential expression *p*-values were calculated using RankProd non-parametric permutation tests. DECs were those with raw back splice junction (BSJ) count across the sample set >10, log_2_FC > 0.5, and *p*-value < 0.05. Heatmap values are the average of biological replicates.

**Figure EV3:**

Screenshot of interactive resource table for transcriptomic data. (**A**) Representative screenshot of the GenePlotter tab where users can query any gene of interest. Supporting datasets were generated for all bulk RNA-Seq data analyzed in this study. Datasets include raw and normalized gene counts. (**B**) Representative screenshot of the CircPlotter tab where users can query any circRNA of interest. Supporting datasets were generated for all bulk RNA-Seq data analyzed in this study. Datasets include raw and normalized BSJ counts.

In primary lytic infections we identified 151, 70, and 316 upregulated (log_2_ fold change (log_2_FC) Infected/Uninfected >0.5) human circRNAs for HSV-1, HCMV, and KSHV, respectively (Fig. 1B,C, Dataset EV7). Four circRNAs were upregulated across viruses (Fig. 1B). A similar number of circRNAs were downregulated after infection (log_2_FC < 0.5), with six overlapping between models (Fig. 1B). In HSV-1 infection, disparate circRNA/mRNA expression changes were particularly evident, resulting in dramatic CIRCscore shifts (Fig. 1C). Multiple loci had CIRCscores >5, indicating the circRNA, rather than the colinear mRNA, was the predominant mature transcript for that gene. An increasing CIRCscore with infection was also present for other lytic infection models (Figs. EV2C and EV1C). We extended our analysis to mouse models, including HSV-1 infected trigeminal ganglia and MHV68 infected cell lines. We identified 113 murine circRNAs upregulated by HSV-1 infection, although none overlapped between latency, explant-induced reactivation, and drug-enhanced reactivation (Fig. EV1B,C, Dataset EV7). There were 72 murine circRNAs upregulated by MHV68 infection, with four overlapping between primary infection and lytic reactivation (Fig. EV2B,C, Dataset EV7). There were 12 circRNAs in common between HSV-1 and MHV68 infection models, of these circMed13l (mmu_circ_0001396) was upregulated in all infection models (Figs. EV1 and EV2).

Differentially expressed circRNAs (DECs) common across disparate virus and cell models hints at a common mode of induction. To investigate this, we performed overrepresentation analysis (ORA) on the colinear genes of circRNAs expressed in human infection models (Appendix Fig. S1). ORA identified enrichment of genes involved in cellular senescence for all fibroblast (MRC-5) models, likely representing that infection is performed in G0 cells. Interestingly, genes within the lysine degradation pathway were enriched after infection of HSV-1, HCMV, and KSHV (Appendix Fig. S1). One biological function of circRNA is regulation of mRNA expression through miRNA sponging (Kristensen et al, 2019). We performed circRNA-miRNA-mRNA network analysis for circRNA commonly upregulated by herpesvirus infection (circEPHB4, circVAPA, circPTK2, and circKMT2C) (Appendix Fig. S2). In silico analysis predicted miRNA-mRNA interaction nodes which were enriched for mRNA involved in adaptive immunity including MHC complex assembly, TAP complex binding, and peptide antigen stabilization, suggesting a potential role in antiviral immunity for these commonly upregulated circRNAs.

### Global distribution shifts for mRNA, lncRNA, and circRNA during lytic infection

**Figure 2:**
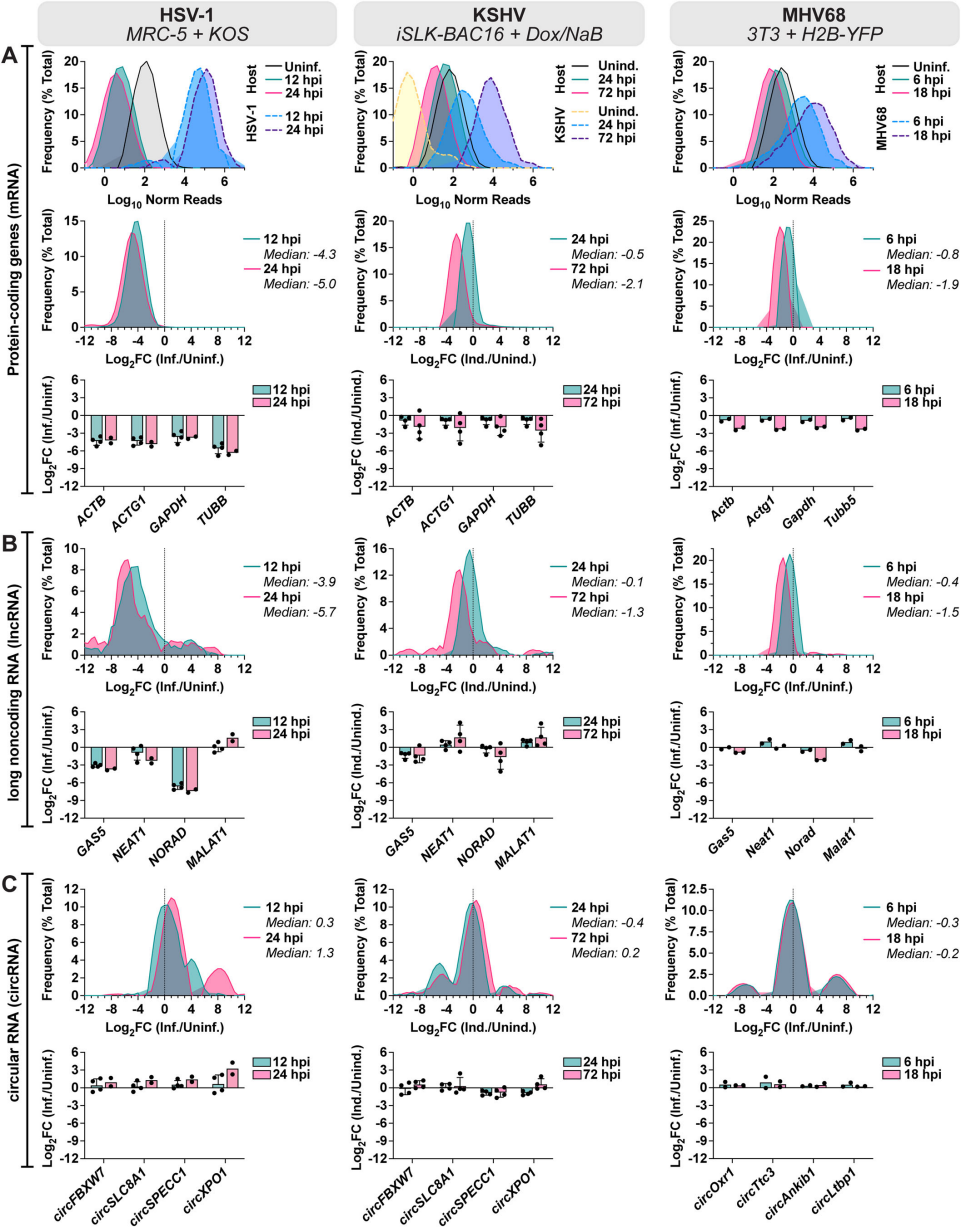
Global distribution shifts for mRNA, lncRNA, and circRNA during lytic infection. (**A**–**C**) Bulk RNA-Seq data from HSV-1 infection (MRC-5 infected with KOS MOI 10, *n* = 2–4), KSHV lytic reactivation (iSLK-BAC16 induced with 1 µg/mL doxycycline 1 mM sodium butyrate, *n* = 4), and MHV68 infection (3T3 infected with H2B-YFP MOI 5, *n* = 2). (**A**) Data for protein-coding genes plotted as relative frequency distribution for log_10_ ERCC normalized reads or log_2_FC (limited to top 10,000 most highly expressed genes). Log_2_FC for representative protein-coding genes is plotted in column bar graphs. (**B**) Data for lncRNA genes plotted as relative frequency distribution for log_10_ normalized reads or log_2_FC (limited to top 100 most highly expressed genes). Log_2_FC for representative lncRNA genes is plotted in column bar graphs. (**C**) Data for circRNA plotted as relative frequency distribution for log_10_ normalized BSJ reads or log_2_FC (limited to top 100 most highly expressed circRNAs). Log_2_FC for representative circRNAs is plotted in column bar graphs. Data Information: Bulk RNA-Seq data was normalized to ERCC spike-in controls, log_2_FC is relative to a paired uninfected or uninduced control. Frequency distribution plots are the average of biological replicates. In column bar graphs, data points are biological replicates, column bars are the average, and error bars are standard deviation.

In HSV-1 infection of MRC-5 (Fig. 1C), circRNA upregulation was at odds with the stark decrease in colinear gene expression. A similar trend was visible, albeit less notable, for other lytic infection models (Figs. EV2C and EV1C). This finding led us to question if circRNAs were resistant to the global downregulation of host RNAs which occurs during lytic infection. Using ERCC normalized RNA-Seq datasets we plotted read distribution shifts for HSV-1, KSHV, and MHV68 lytic infection (Fig. 2). We compared expression changes for protein-coding genes (mRNA), long noncoding RNAs (lncRNAs), and circRNAs. In Fig. 2A we observed a sharp decrease in host mRNA levels, with a median log_2_FC of −4.3 (HSV-1 12 hpi), −2.2 (KSHV 72 hpi), and −1.9 (MHV68 18 hpi). This coincides with high levels of viral gene expression. As has been previously reported (Abernathy et al, 2014; Covarrubias et al, 2011; Gaglia et al, 2012), this effect was partially ablated for lncRNAs with median log_2_FC of −3.9 (HSV-1 12 hpi), −1.3 (KSHV 72 hpi), and −1.5 (MHV68 18 hpi) (Fig. 2B). Strikingly, host circRNAs were globally resistant to HSV-1 shutoff and instead exhibited a general upregulation (log_2_FC + 1.3) by 24 hpi (Fig. 2C). During KSHV and MHV68 infection, host circRNA expression changes were tri-modal with a downregulated, unaffected, and upregulated subpopulation. However, the bulk of circRNA species were again largely unchanged with median log_2_FC of +0.2 (KSHV 72 hpi) and −0.2 (MHV68 18 hpi). We examined if the tri-modal distribution of host circRNAs during KSHV reactivation may be explained by sequence differences. A degenerate motif “UGAAG” can increase substrate recognition for the KSHV endoRNase, SOX (Gaglia et al, 2015). We posited that underrepresentation of this motif in circRNAs may allow them to escape KSHV host shut off. We performed motif scanning (Table 1) for our tri-modal circRNA populations to assess incidence of the motif. Proportions of motif-harboring circRNAs did not correlate with up- or down-regulation upon reactivation. Overall density of degenerate motifs was, however, highest in downregulated circRNAs and lowest in upregulated circRNAs, suggesting a potential role of UGAAG frequency in SOX resistance. Our analysis demonstrates that host circRNAs, as a species of RNAs, are resistant to the global downregulation of RNA which occurs during lytic herpesvirus infection.

**Table 1:** Analysis of SOX substrate-targeting motif.

Subset: Log_2_FC (3 dpi/Uninduced	# circRNA	# circRNA in circAtlas	# UGAAG motifs	# Motifs per circRNA	# Motifs per 1000 nt	UGAAG+ circRNA	
						# circRNA	% Total
Upregulated: ≥0.5	42	39 circRNA (22,459 nt)	71	1.9	3.2	37	88%
Unchanged: <0.5 and >−0.5	28	28 circRNA (20,975 nt)	95	3.7	4.5	26	93%
Downregulated: ≤−0.5	30	25 circRNA (12,900 nt)	104	4.7	8.1	22	73%

The presence of SOX substrate-targeting motifs “UGAAG” was determined for subsets of human circRNAs from our KSHV reactivation model (iSLK-BAC16).

### CircRNAs are resistant to viral endonuclease-mediated decay

**Figure 3:**
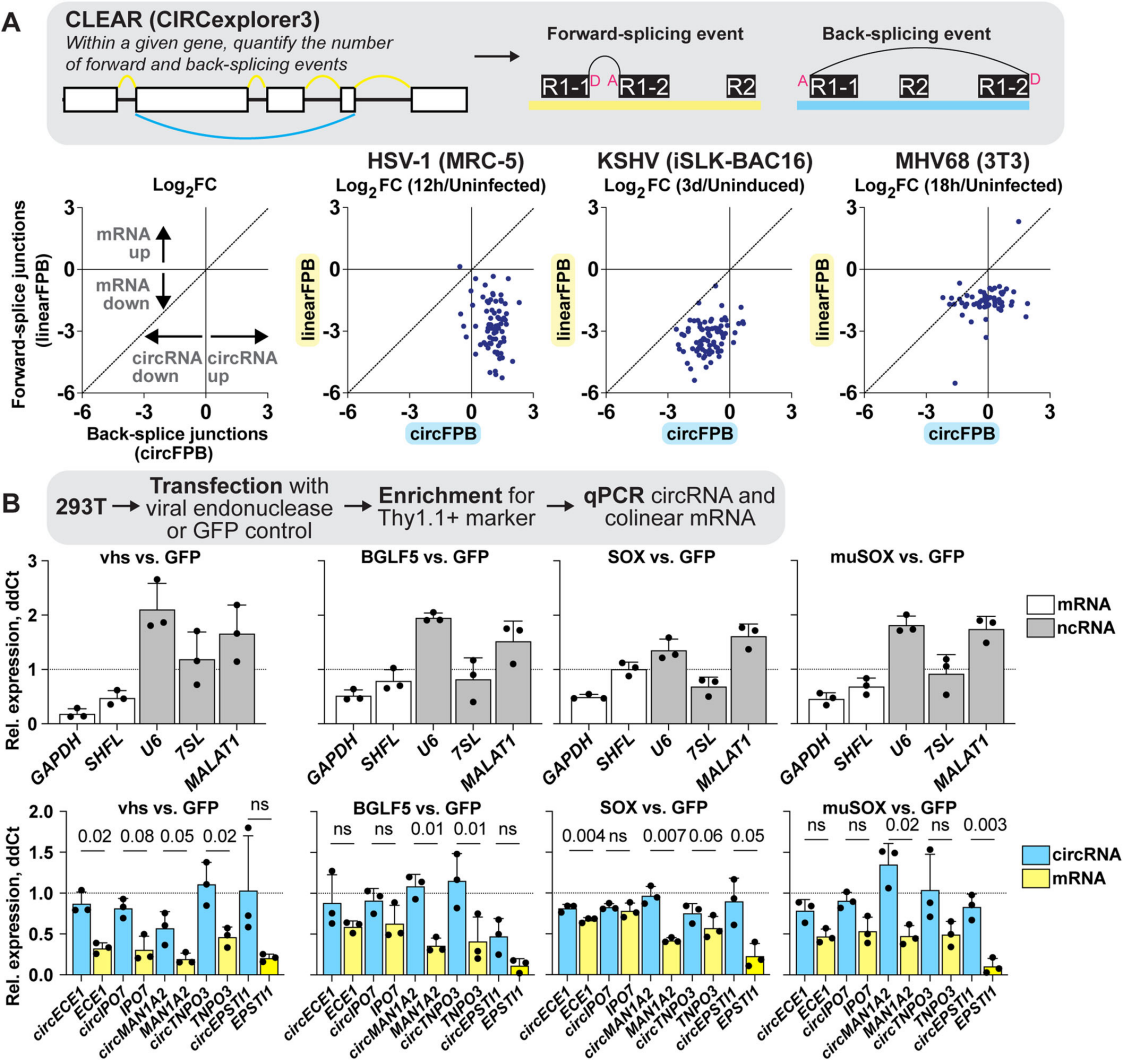
CircRNA are resistant to viral endonuclease-mediated decay. (**A**) Bulk RNA-Seq data from HSV-1 (MRC-5 infected with KOS MOI 10 for 12 h, *n* = 4), KSHV (iSLK-BAC16 reactivated with Dox/NaB for 3 days, *n* = 4), and MHV68 (3T3 infected with H2B-YFP MOI 5 for 18 h, *n* = 2) infection. Graphs are limited to genes where raw BSJ and forward splice junction (FSJ) counts were >1 across all biological replicates. Each dot is the average log_2_FC of biological replicates, with each dot representing linearFPB and circFPB for a distinct gene. (**B**) Ectopic expression assays of viral endonucleases (vhs, BGLF5, SOX, muSOX) versus control (GFP) vector in 293T cells. RNA was collected after 24 h and transcripts quantified by qPCR (*n* = 3). Data is plotted as relative expression (ddCt) using 18S rRNA as the reference gene, and relative to a paired GFP transfected sample. Data points are biological replicates, column bars are the average, and error bars are standard deviation. Data Information: Paired t-tests were performed comparing circRNA and colinear mRNA expression, *p*-values < 0.1 are labeled and not significant (n.s.) indicates *p*-values ≥ 0.1. Source data are available online for this figure.

Figure 2 plots expression changes across an entire RNA class. To investigate if similar trends occurred for circRNAs and mRNAs derived from the same gene, we evaluated expression shifts for circRNAs (circFPB) and mRNAs (linearFPB) as log_2_FC (Infected/Uninfected) for HSV-1, KSHV, and MHV68 lytic infection (Fig. 3A). Each dot is a gene that can be alternatively spliced, generating both circRNAs and mRNAs. Echoing our results in Fig. 1C and Fig. 2A,C, circRNA abundance increased while mRNA decreased for a vast majority of genes after HSV-1 infection (Fig. 3A). For KSHV and MHV68, mRNA downregulation was consistently more pronounced than circRNA downregulation. Bulk RNA-Seq examines steady-state transcript abundance, averaging the effects of transcriptional activity in addition to co- and post-transcriptional processing such as splicing and decay. To examine what most influences circRNA upregulation we examined our previously published nascent RNA-Seq and ChIP-Seq data (Data ref: Dremel and DeLuca 2019; Data ref: Dremel et al, 2022a; Data ref: Dremel et al, 2022b; Data ref: Dremel et al, 2022c) for a subset of genes colinear to circRNAs upregulated during HSV-1 infection (Appendix Fig. S3). All four genes (*POLR2A, EPHB4, CREBBP, PLEKHM1*) had a drop in RNA Polymerase II (Pol II) and TATA-binding protein (TBP) occupancy by 4 hpi, consistent with published mechanisms of host transcriptional shutoff during HSV-1 infection (Abrisch et al, 2015; Birkenheuer et al, 2018; Dremel and DeLuca, 2019; McSwiggen et al, 2019). In the case of *EPHB4*, *CREBBP*, and *PLEKHM1* we also observed a drop in 4 thiouridine (4sU)-Seq read coverage, consistent with decreased nascent transcripts. This data suggests circRNA expression changes—for at least this subset—are related to co- or post-transcriptional processing.

Given this observation, we explored if differences in viral endoRNase-mediated decay might explain the disparate expression profiles for circRNA and their colinear genes. Using the approach of Rodriguez et al (2019), plasmids expressing HSV-1 vhs, EBV BGLF5, KSHV SOX, and MHV68 muSOX were transfected into 293T cells for 24 h. Ectopic gene expression and Thy1.1 surface expression were confirmed by RT-qPCR and flow cytometry, respectively (Appendix Fig. S4). Transcript levels were quantified relative to a paired GFP vector control (Fig. 3B). As expected, transfection with viral endoRNases decreased *GAPDH* expression (Rodriguez et al, 2019). Conversely, ncRNAs such as *U6 snRNA*, *7SL*, and *MALAT1* were either unaffected or were increased. We also recapitulated prior findings, regarding escape of the *SHFL* mRNA from SOX-mediated decay (Rodriguez et al, 2019). We then tested expression changes of circRNAs and colinear mRNAs using divergent or convergent primers, respectively. Across all genes tested and viral endoRNases transfected, circRNA were more resistant to decay as compared to their colinear gene product. CircRNA and colinear mRNA expression differed most significantly in vhs and SOX transfected 293T (Fig. 3B). Thus, we propose circRNAs as a general class of host shutoff escapees. Our data agrees with prior work from HSV-1 (Friedl et al, 2022), arguing circRNA resistance to viral endoRNases is primarily responsible for the disparate expression changes of circRNAs relative to their colinear mRNA gene products.

### Detection of interferon-stimulated circRNAs (ISCs)

**Figure EV4:**
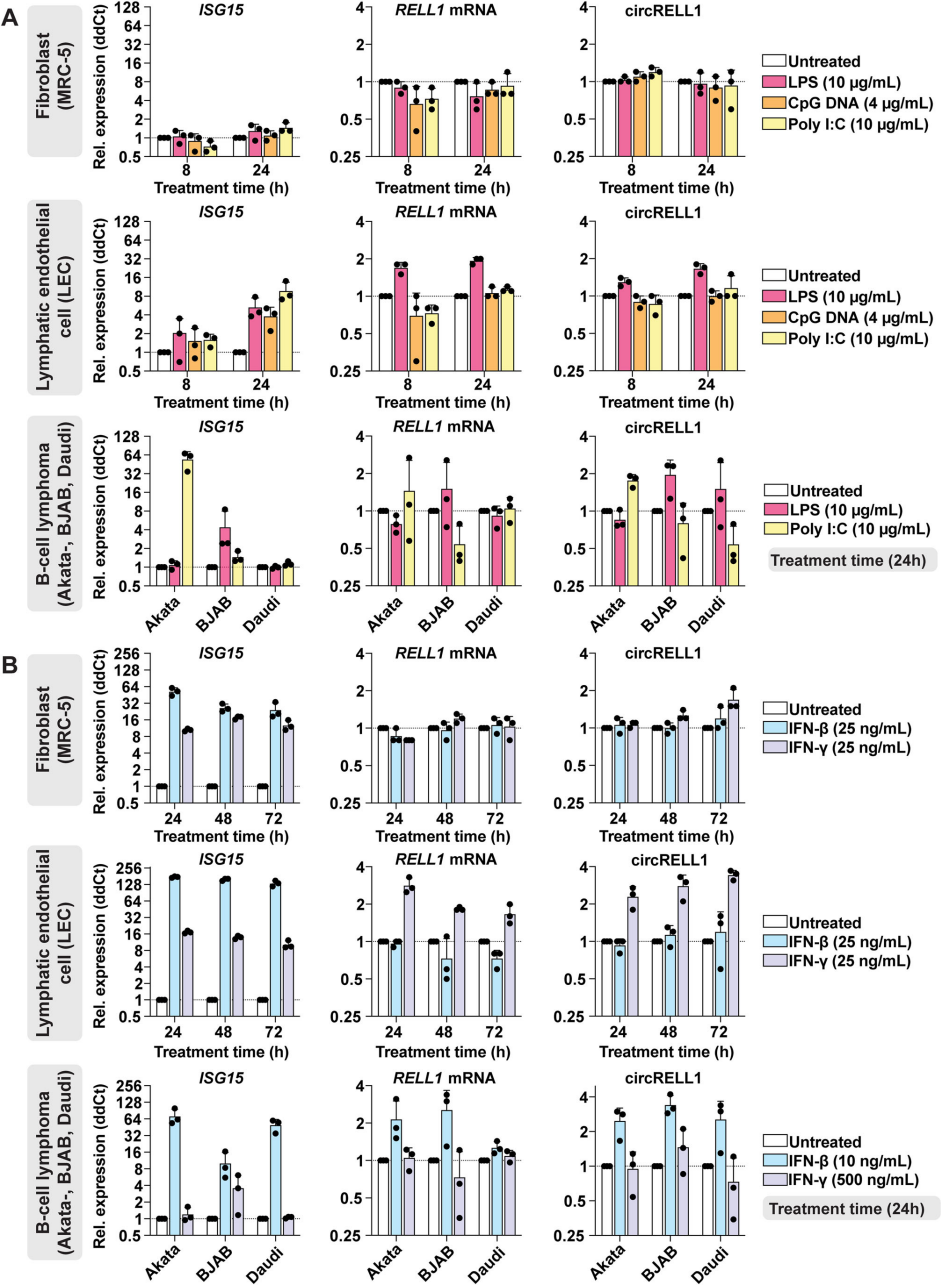
CircRELL1 expression changes in response to immune stimuli. (**A**) Transcript quantitation for RNA collected from fibroblasts (MRC-5), lymphatic endothelial cells (LEC), or B-cell lymphoma cells (Akata-, BJAB, Daudi) treated with lipopolysaccharide (LPS), poly I:C, or CpG DNA (*n* = 3). (**B**) Transcript quantitation for RNA collected from MRC-5, LEC, or B-cell lymphoma cells treated with IFN-β and -γ (*n* = 3). Data Information: qPCR data is plotted as relative expression (ddCt) using 18S rRNA as the reference gene, and relative to a paired untreated sample. Data points are biological replicates, column bars are the average, and error bars are standard deviation.

The most significantly upregulated pathways during HSV-1, HCMV, and KSHV infection fell largely within the category of immune responses, with IFN-β and -γ predicted as upstream regulators (Appendix Fig. S5). This led us to investigate if circRNA expression may be modulated by innate immune signaling. We treated fibroblast (MRC-5), lymphatic endothelial cell (LEC), and B-cell lymphomas (Akata-, BJAB, Daudi) with immune stimulants including lipopolysaccharide (LPS), CpG DNA, poly I:C, IFN-β and -γ (Fig. EV4). A canonical interferon-stimulated gene (ISG), *ISG15*, was measured as a surrogate for immune stimulation. We measured circRELL1 expression, as this circRNA was upregulated in HCMV, KSHV, and to a lesser extent, HSV-1 infection (Fig. 1C). circRELL1 was upregulated in LEC treated with LPS or IFN-γ and B-cell lymphomas treated with poly I:C, LPS, or IFN-β (Fig. EV4). *ISG15* activation did not always correlate with circRELL1 expression—notably CpG and poly I:C treatment largely failed to induce expression. In addition, IFN-β and -γ caused inverse phenotypes in circRELL1 expression when comparing LEC and B-cell models (Fig. EV4B). These findings demonstrate that circRELL1 can be upregulated by Toll-like receptor engagement and type I and II interferon stimulation, and the expression profile varies by cell type and mechanism of immune stimulation.

**Figure 4:**
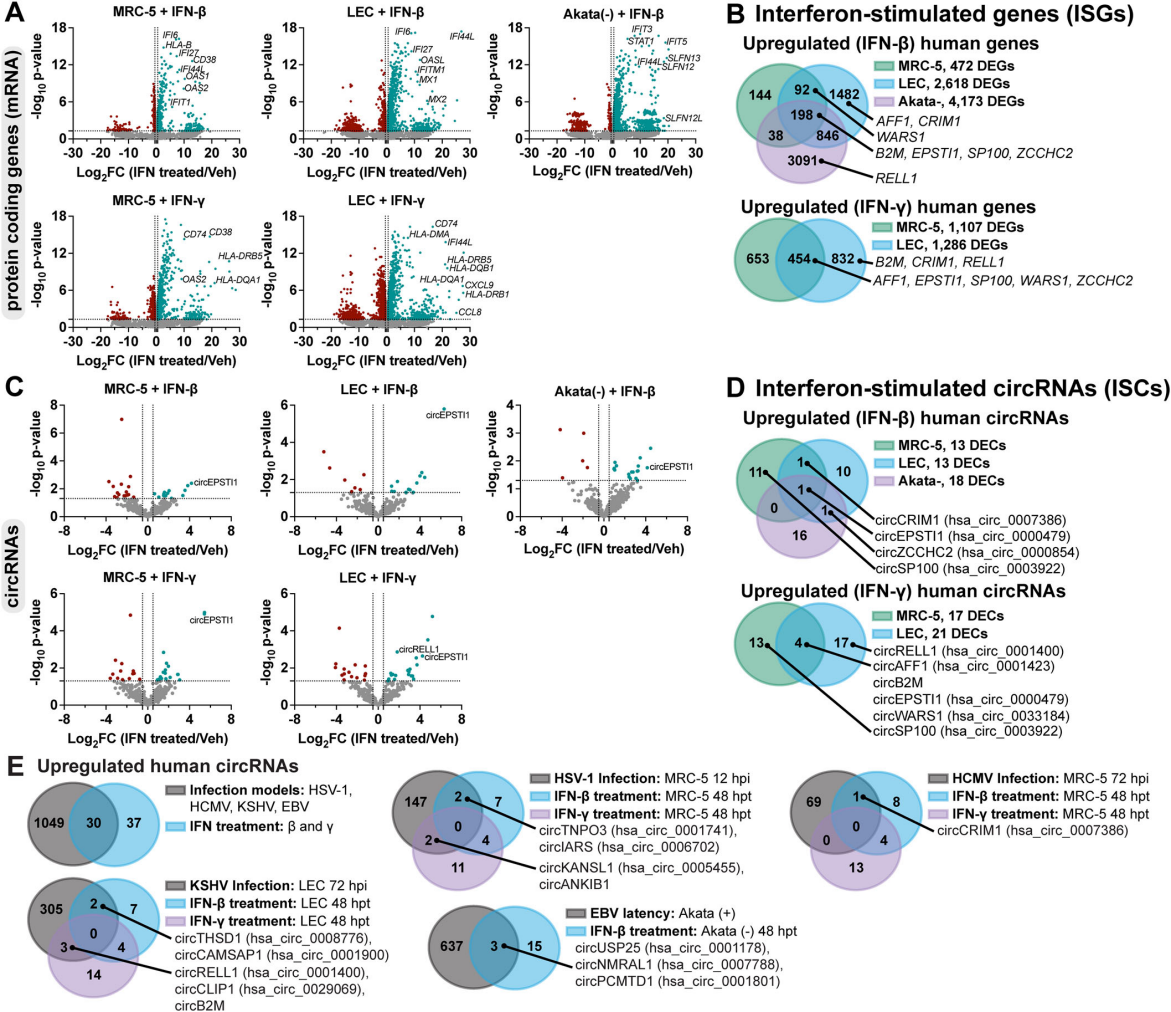
Detection of interferon-stimulated circRNAs (ISCs). (**A**–**E**) Bulk RNA-Seq data from MRC-5, LEC, or Akata- cells were treated with recombinant interferons for 48 h (*n* = 3). MRC-5 and LEC were treated with IFN-β and -γ (25 ng/mL conc). Akata- were treated with IFN-β (10 ng/mL). (**A**,**C**) Volcano plots for normalized protein-coding gene (mRNA) or circRNA reads. (**B**,**D**) Venn diagrams of significantly upregulated circRNA or mRNAs. (**E**) Overlap of circRNAs upregulated during herpesvirus infection (Fig. 1) or interferon-stimulation. Data Information: Bulk RNA-Seq data was normalized to ERCC spike-in controls. Log_2_FC was calculated relative to a paired untreated sample. Adjusted *p*-values (Benjamini and Hochberg) were calculated using EdgeR quasi-likelihood F test method. Differentially expressed genes or circRNAs were those with raw counts across the sample set >10, log_2_FC > 0.5, and *p*-value < 0.05.

To globally profile interferon-stimulated circRNAs (ISCs) we performed RNA-Seq on fibroblast, lymphatic endothelial, and B-cells treated with IFN-β and -γ. B-cells (Akata) were only treated with IFN-β as we found them refractory to IFN-γ (Fig. EV4B). Transcriptomic analysis identified strong upregulation of many canonical ISGs including *OAS1*, *OAS2*, *OASL*, *IFIT1*, *IFITM1*, *MZ1*, *HLA*-*DRB1*, *HLA*-*DQA1*, and *HLA*-*DMA* after IFN treatment (Fig. 4A, Dataset EV6). The extent and range of ISGs was most pronounced for B-cells treated with IFN-β (*n* = 4173 DEGs) (Fig. 4A,B) and smallest for fibroblast treated with IFN-β (*n* = 472 DEGs). We identified approximately a dozen interferon-stimulated circRNAs in each model (Fig. 4C). Of these, circEPSTI1 (hsa_circ_0000479) was upregulated in all conditions (Fig. 4D). We tested if circRELL1 and circEPSTI1 expression changes could be recapitulated in peripheral blood mononuclear cells (PBMCs) and observed a 1.5 and 9-fold increase, respectively, after IFN-β treatment (Appendix Fig. S6).

**Figure EV5:**
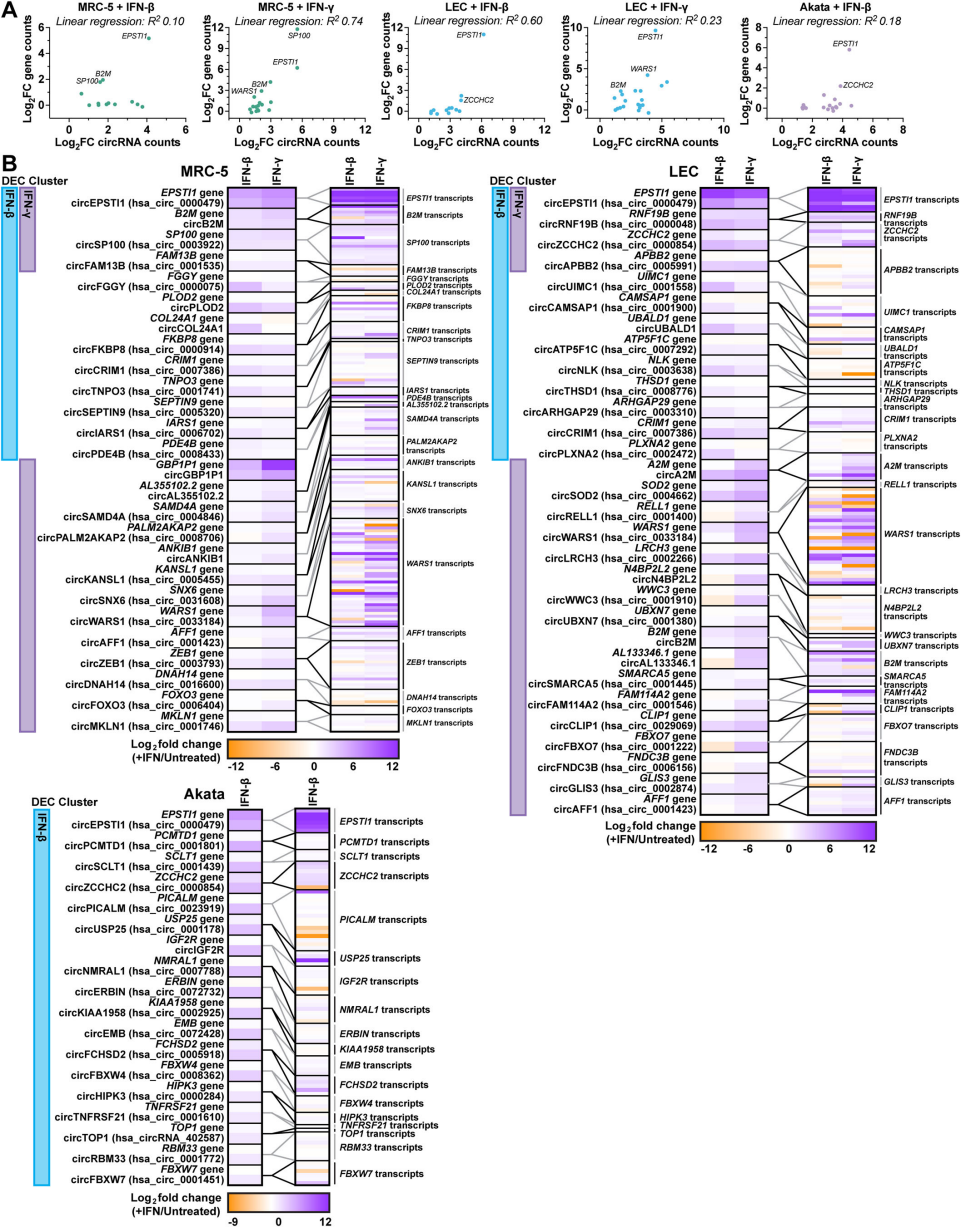
Transcript isoform analysis of interferon-stimulated cells. (**A**) Bulk RNA-Seq data from Fig. 4 plotting log_2_FC (+IFN/untreated) for DECs relative to their colinear gene reads. Genes with similar levels of upregulation for circRNA and colinear gene species are labeled. (**B**) Bulk RNA-Seq data from Fig. 4 plotted as heatmaps showing log_2_FC (+IFN/untreated) for circRNA species and their colinear gene or transcript isoforms. Gene and transcript quantitation was performed using Salmon and a reference transcriptome fasta. The sample (+IFN-β or +IFN-γ) where a differentially expressed circRNA (DEC) was identified are labeled on the left of each heatmaps as “DEC cluster”. Data Information: Bulk RNA-Seq data was normalized to ERCC spike-in control and log_2_FC was determined relative to a paired untreated sample. All data plotted is the average of biological replicates (*n* = 3). The relationship between circRNA and colinear gene expression changes was assessed by linear regression analysis, R^2^ values are given.

A number of colinear mRNAs and circRNAs were stimulated by interferon treatment, including transcripts derived from *CRIM1*, *EPSTI1*, *ZCCHC2*, *SP100*, *AFF1*, *B2M*, and *WARS* (Fig. 4B,D). The typical mechanism of ISG induction relies upon promoter activation by factors like IRF3, IRF7, IRF9, STAT1, and STAT2. If this mechanism is similarly responsible for circRNA upregulation, we would expect circRNA levels to correlate with changes in their colinear gene products. We plotted gene expression relationship for ISCs and colinear transcripts (Fig. EV5). A subset of ISC-hosting genes, *EPSTI1*, *SP100*, *B2M*, and *WARS*, had a direct relationship between circRNA and colinear gene expression changes. However, this was not globally the case, with linear regression R^2^ values ranging from <0.3 (MRC-5 + IFN-β, LEC + IFN-γ, Akata + IFN-β) to ≥ 0.6 (MRC-5 + IFN-γ, LEC + IFN-β) (Fig. EV5A). We next examined overlaps between our interferon-stimulated and infection-stimulated circRNAs (Fig. 1, Dataset EV1, EV2, EV3, and EV6). EBV-stimulated circRNAs (Fig. 4E) were from our previously published microarray dataset which compared EBV-positive versus EBV-negative Akata cells (Data ref: Tagawa et al, 2023). Approximately half of the ISCs detected were also upregulated during models of HSV-1, HCMV, KSHV, or EBV infection (Fig. 4E). These findings demonstrate tunability in circRNA expression with dependence on cell type and immune stimulation.

### circRELL1 restricts HSV-1 lytic infection

**Figure 5:**
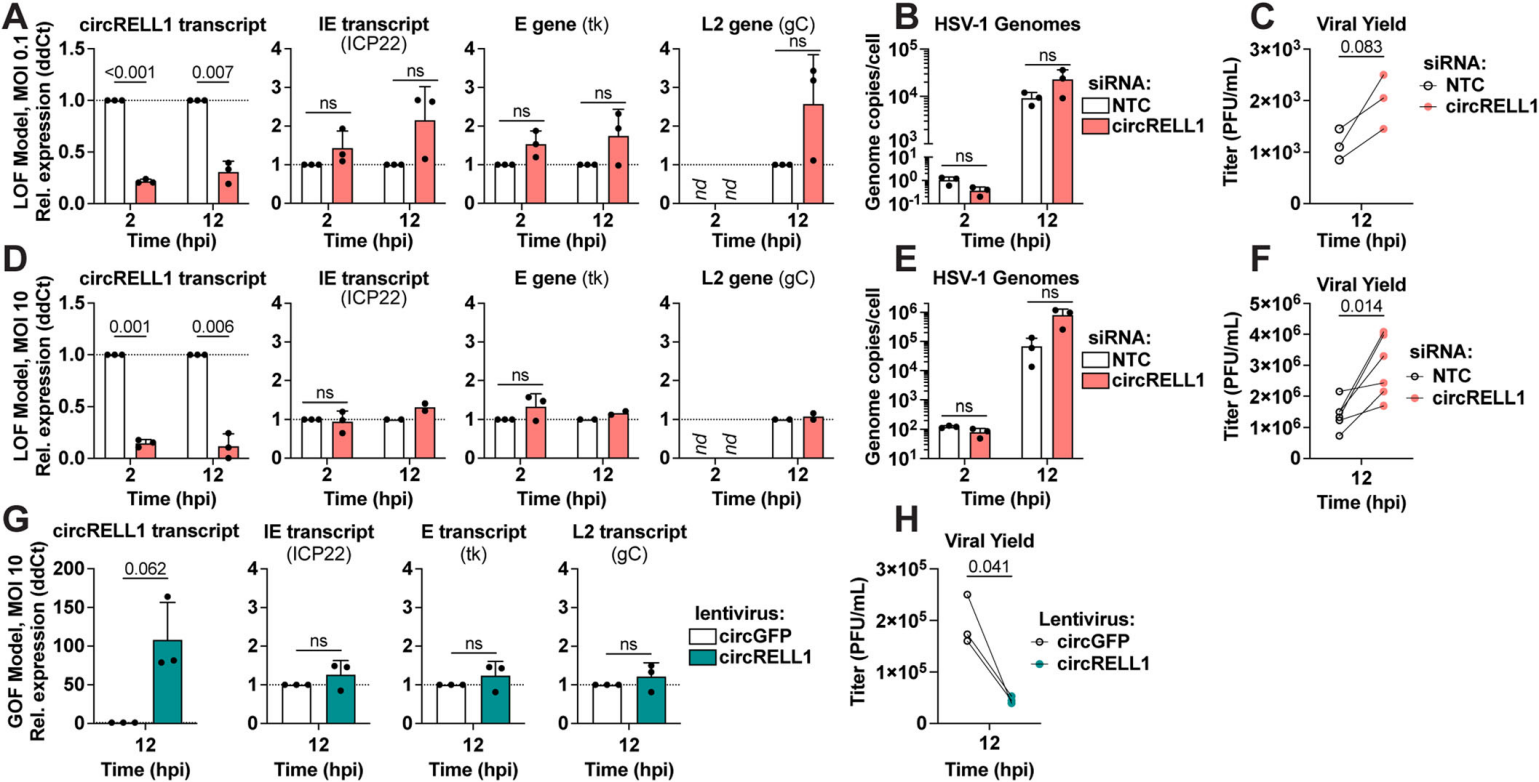
circRELL1 (hsa_circ_0001400) restricts HSV-1 lytic infection. (**A**–**F**) circRELL1 was depleted in MRC-5 cells using siRNAs for 48 h and subsequently infected with HSV-1 strain KOS at MOI of 0.1 or 10 PFU/cell. Data is relative to a paired Non-Targeting Control siRNA (NTC). (**G**,**H**) MRC-5 cells were infected with a lentivirus expressing circRELL1 for 48 h and subsequently infected with HSV-1 strain KOS at MOI of 10 for 12 h. Data is relative to a control lentivirus expressing circGFP. (**A**,**D**,**G**) RNA was collected from the cell fraction and reverse transcribed. qPCR data is plotted as relative expression (ddCt) using 18 S rRNA as the reference gene (*n* = 2–3). (**B**,**E**) DNA was isolated from the cell fraction and assessed by qPCR for viral and host genome copies (*n* = 3). (**C**,**F**,**H**) Supernatant was collected at 12 hpi and assessed by plaque assay. Data points (**C**,**H***n* = 3; **F***n* = 6) are biological replicates, column bars are the average, and error bars are standard deviation. Data Information: Paired two-tailed t-tests were performed, *p*-values < 0.1 are labeled and not significant (n.s.) indicates *p*-values ≥ 0.1. Source data are available online for this figure.

In this study, we found circRELL1 to be induced by HSV-1 (1.3-fold), HCMV (1.4-fold), and KSHV (2-fold) infection (Fig. 1C). circRELL1 was also upregulated by immune stimulants including LPS, poly I:C, IFN-β and -γ (Fig. EV4, Appendix Fig. S6). Our lab has previously reported inhibition of lytic KSHV infection by circRELL1 (Tagawa et al, 2023). Thus, we questioned if it could be broadly antiviral, by perturbing circRELL1 within the context of HSV-1 infection. To test the impact of loss of function, we depleted circRELL1 48 h prior to infection using siRNAs targeting the BSJ. Our siRNAs were specific to the circRNA species with the colinear mRNA, *RELL1*, unaffected by siRNA treatment (Appendix Fig. S7). MRC-5 cells were infected at a low (0.1 plaque forming units (PFU)/cell) and high (10 PFU/cell) multiplicity of infection (MOI). We achieved significant depletion (4- to 10-fold decrease) of circRELL1 as compared to a Non-Targeting Control (Fig. 5A,D). There was no significant change in viral gene expression for immediate early (IE), early (E), and true late (L2) transcripts after circRELL1 knockdown. Viral entry was similarly unaffected with the level of viral genomes at 2 hpi comparable between Non-Targeting Control and siRNA against circRELL1 (Fig. 5B,E). The effect of circRELL1 depletion was most apparent when measuring infectious viral yield, with a 1.8- and 2.2-fold increase for low and high MOI infections, respectively (Fig. 5C,F). For gain of function studies, we transduced circRELL1 in MRC-5 with a replication-null lentivirus for 48 h followed by infection with HSV-1 at high MOI (10 PFU/cell). By 60 h post-lentivirus infection, circRELL1 expression was 100-fold greater than our lentivirus control that harbors circGFP (Fig. 5G). While we observed no alterations in IE, E, or L2 viral transcripts, viral yield was decreased 4.4-fold by circRELL1 overexpression (Fig. 5G,H). Our loss and gain of function models agree, supporting an anti-lytic role for circRELL1 in HSV-1 infection that appeared to be independent of viral gene expression.

## Discussion

**Figure 6:**
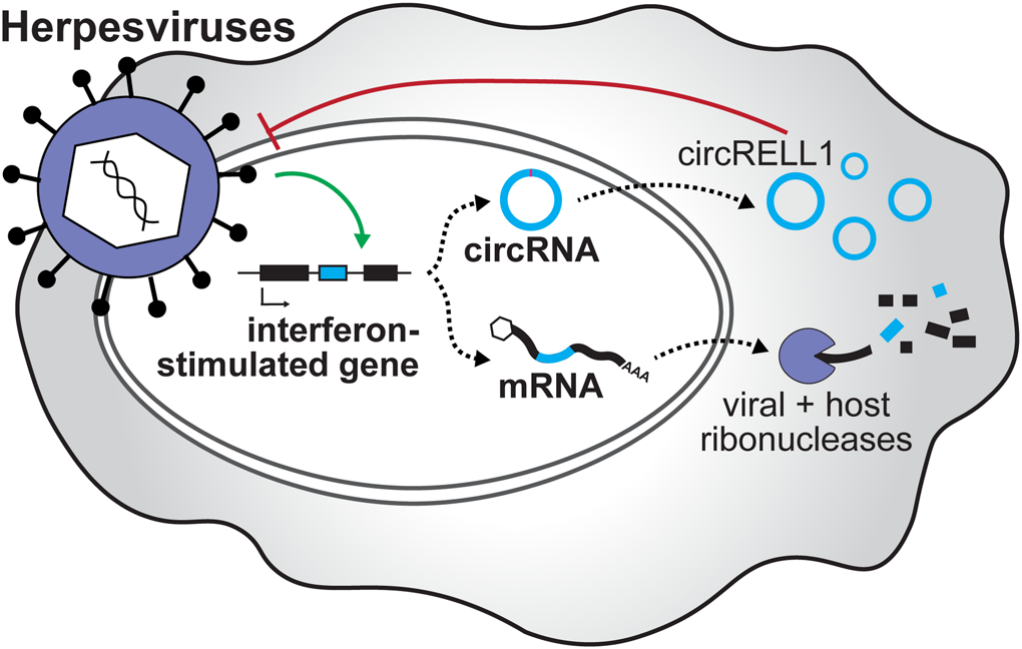
Proposed polycistronic model for interferon-stimulated genes. We propose a polycistronic model in which interferon-stimulated genes can produce both mRNA and circRNA with antiviral activity. This is critical in cases of host shut off, such as alpha- and gamma-herpesvirus infection, where the mRNA product is degraded but circRNA escapes. The interferon-stimulated circRNA, circRELL1, exemplifies this model. EBV, KSHV, and HCMV infection upregulates circRELL1 expression which functions to suppress lytic infection of HSV-1 and KSHV.

CircRNAs are gaining traction as important factors at the virus-host interface. CircRNAs have an interesting combination of physical features: versatility to interact with DNA, RNA, and proteins simultaneously, longer half-lives than colinear mRNAs, and secretion to extracellular spaces. Some of these features are shared by other molecules like lncRNAs or microRNAs, but circRNAs uniquely possess all those flexible and multi-faceted characteristics. In this study, we probed the role of circRNAs during herpesvirus infection. We performed comparative circRNA expression profiling and identified four host circRNAs commonly upregulated by all subfamilies of human herpesviruses (Fig. 1). Whether there are universal functions of these commonly regulated circRNAs is not clear but overrepresentation analysis of colinear transcripts showed their enrichment in cell division/senescence pathways and lysine degradation (Appendix Fig. S1). In silico circRNA-miRNA-mRNA interaction networks predict circEPHB4, circVAPA, circPTK2, and circKMT2C may regulate cellular immunity via repression of miRNA-mediated decay of mRNAs involved in antigen presentation (Appendix Fig. S2). We identified a subset of circRNAs induced by herpesvirus infection and interferon stimulation (Fig. 4). Since circRNAs and mRNAs expressed from the same loci can have distinct targets and functions, they can be thought of as polycistronic genes. We propose a two-pronged model in which interferon-stimulated genes may encode both mRNA and circRNA with immune-regulatory activity (Fig. 6). This is critical in cases of host shutoff, such as alpha- and gamma-herpesvirus infection, where the mRNA product is degraded but circRNA escapes.

CircRNAs were resistant to ectopic expression of HSV-1, EBV, KSHV, and MHV68 endoribonucleases (Fig. 3B), suggesting them to be a general class of host shutoff escapees. These results were echoed during lytic infection, with global circRNA levels generally unaffected as lytic infection progressed (Fig. 2C). For the gamma-herpesviruses, KSHV and MHV68, we observed a subpopulation of circRNA which were downregulated by infection. This created a tri-modal circRNA distribution which was not observed for HSV-1. The different circRNA expression modalities for alpha-versus gamma-herpesviruses likely reflects the mechanisms by which specific endoRNases are recruited. HSV-1 vhs targets the 5′ untranslated region (UTR) of mRNAs near the 5′ cap and binding is mediated by the translation factors eIF4H and eIF4AI/II (Feng et al, 2005; Karr and Read, 1999; Page and Read, 2010; Shiflett and Read, 2013). CircRNAs may escape vhs degradation as they do not have a 5′ cap or UTR. This mechanism would not apply to gamma-herpesvirus endoRNases (BGLF5, SOX, muSOX) which bind to a degenerate motif with little preference for relative position within the transcript (Abernathy and Glaunsinger, 2015; Gaglia et al, 2015). Motif scanning suggested high frequency of the consensus “UGAAG” may be partially responsible for host circRNA up- or down-regulation during KSHV lytic reactivation (Table 1). This, however, does not rule out the possibility that SOX targeting may be ameliorated by additional factors. An RNA motif called the SOX-resistant element (SRE) can confer resistance to cleavage in a cis-acting, dominant fashion (Hutin et al, 2013; Muller and Glaunsinger, 2017). CircRNAs tend to shape characteristic stem structures which may potentially act as SREs (Liu et al, 2019). RNA modifications or general accessibility may also confer endoRNase resistance. The host is known to use m6A modifications to distinguish self and non-self circRNA (Chen et al, 2019). Recently, a m6A reader *YTHDC2* was found to mediate resistance to KSHV SOX (Macveigh-Fierro et al, 2022). RNA Binding Proteins (RBPs) may also play a role in circRNA decay as they can aggregate and cover up to 100% of circRNAs (Okholm et al, 2020), potentially restricting endoRNase substrate recognition. A combination of circRNA sequence and structure likely contributes to the subsets of gamma-herpesvirus endoRNase susceptibility.

CircRNA upregulation in disparate virus and cell models hints at a common mode of induction. As a first line of the antiviral response, the host senses and responds to pathogens through pattern recognition receptors to induce interferons and interferon-stimulated genes. In line with this, the most significantly upregulated pathways during lytic infection were immune-related with IFN-β and -γ predicted as upstream regulators (Appendix Fig. S5). We profiled expression after type I and II interferon treatment and identified 67 upregulated circRNAs (Fig. 4), with half of these also upregulated in our infection models. mRNA and circRNA products for several genes including *EPSTI1*, *B2M*, and *ZCCHC2* were induced comparably after interferon stimulation. These interferon-stimulated circRNAs are therefore likely regulated at the level of gene expression. ISCs with disparate expression changes to their colinear gene product (Fig. EV5A), may be the results of secondary effects such as altered back-splicing or decay. Zinc finger antiviral protein (ZAP) is upregulated upon IFN stimulation and recruits de-capping enzyme Dcp1 and deadenylase PARN to degrade mRNAs (Abernathy and Glaunsinger, 2015). circRNAs are devoid of cap structures or accessible poly(A) tails and likely to resistant to ZAP-mediated degradation, which may result in the accumulation of circRNAs compared to colinear mRNAs upon IFN stimulation.

circRELL1 exemplifies the functionally conserved circRNA. circRELL1 expression was induced by recombinant interferons (β and γ) and the pathogen-associated molecular pattern (PAMP), LPS. Of the stimuli tested, circRELL1 expression was largely unaffected by poly I:C and CpG treatment (Fig. EV4). Poly I:C and CpG DNA are recognized by endosomal toll-like receptors (TLR), TLR3 and TLR9, whereas LPS is sensed by TLR4 on the cell surface. The subcellular localization of TLRs and relative amplitude of their downstream signaling may impact circRELL1 upregulation. In addition, poly I:C treatment can induce robust RNase L-dependent circRNA decay (Liu et al, 2019); a phenomenon that may disguise poly I:C dependent circRELL1 upregulation. We also observed upregulation of circRELL1 following beta- (HCMV) and gamma-herpesvirus (EBV, KSHV) infection. We previously showed that circRELL1 has an anti-lytic cycle role during infection with a gamma-herpesvirus, KSHV (Tagawa et al, 2018; Tagawa et al, 2023). Here, comparable defects in infectious progeny after circRELL1 perturbation were observed for the alpha-herpesvirus, HSV-1 (Fig. 5). This functional conservation signifies the importance of commonly regulated circRNAs identified in this study. The mechanism by which this circRNA represses lytic infection is not fully understood. circRELL1 was found to interact with *TTI1* mRNA, a component of mTOR complex, which is targeted by EBV, KSHV, HCMV, and HSV-1 to regulate viral replication (Le Sage et al, 2016). Effects of infection-induced circRNAs on mTOR signaling pathway and its consequences thus warrant further study.

## Methods

### Reagents and tools

See Table 2.

**Table 2:** Reagents and tools.

Reagent/Resource	Reference or source	Identifier or catalog number
**Experimental models**		
Vero cells (*C. aethiops*)	ATCC	CCL-81
MRC-5 cells (*H. sapiens*)	ATCC	CCL-171
A20 HE-RIT (*M. musculus*)	Santana et al, 2017*Pathogens*	N/A
iSLK-BAC16 cells (*H. sapiens*)	Brulois et al, 2012 *Journal of Virology*	N/A
293T cells (*H. sapiens*)	ATCC	CRL-3216
Akata, EBV-negative (-) cells (*H. sapiens*)	Komano et al, 1998 *Journal of Virology*	N/A
Daudi cells (*H. sapiens*)	ATCC	CCL-213
BJAB cells (*H. sapiens*)	DSMZ	ACC757
HDLEC cells (*H. sapiens*)	PromoCell	C-12216
BALB/cAnNTac (*M. musculus*)	Taconic Biosciences	N/A
**Recombinant DNA**		
pCMV-Thy1.1-F2A-dsGFP	Rodriguez et al, 2019*Journal of Virology*	N/A
pCMV-Thy1.1-F2A-vhs	Rodriguez et al, 2019*Journal of Virology*	N/A
pCMV-Thy1.1-F2A-BGLF5	Rodriguez et al, 2019*Journal of Virology*	N/A
pCMV-Thy1.1-F2A-SOX	Rodriguez et al, 2019*Journal of Virology*	N/A
pCMV-Thy1.1-F2A-muSOX	Rodriguez et al, 2019*Journal of Virology*	N/A
**Antibodies**		
Mouse monoclonal anti-Thy1.1, Alexa Fluor 647 conjugated	BioLegend, clone OX-7	202526
**Oligonucleotides and other sequence-based reagents**		
GAPDH_F	IDT	CAGAACATCATCCCTGCCTCTACT
GAPDH_R	IDT	GCCGAGCTTCCCGTTCA
UL23 (tk)_F	IDT	ACCCGCTTAACAGCGTCAACA
UL23 (tk)_R	IDT	CCAAAGAGGTGCGGGAGTTT
US1 (ICP22)_F	IDT	TTTGGGGAGTTTGACTGGAC
US1 (ICP22)_R	IDT	CAGACACTTGCGGTCTTCTG
UL44 (gC)_F	IDT	GTGACGTTTGCCTGGTTCCTGG
UL44 (gC)_R	IDT	GCACGACTCCTGGGCCGTAACG
GFP_F	IDT	GAAGCAGCACGACTTCTTCAA
GFP_R	IDT	AGTCGATGCCCTTCAGCTC
vhs_F	IDT	ATCCAACACAATATCACAGCCCATCAACAG
vhs_R	IDT	CGCCAACCTCTATCACACCAACACG
BGLF5_F	IDT	GACCTCTTGCATGGCCTCTT
BGLF5_R	IDT	GTGCCGCTTCAAGTACCTCT
SOX_F	IDT	TGGGCGAGTTTATTGGTAGTGAGG
SOX_R	IDT	CTCCACTAGACAGCAGATGTGG
muSOX_F	IDT	CCCTGGACACTGCTTTCAAT
muSOX_R	IDT	GCCTCACAGGGGTTTTTGTA
18S rRNA_F	IDT	GTAACCCGTTGAACCCCATT
18S rRNA_R	IDT	CCATCCAATCGGTAGTAGCG
ISG15_F	IDT	GAGAGGCAGCGAACTCATCT
ISG15_R	IDT	CTTCAGCTCTGACACCGACA
U6_F	IDT	GGAATCTAGAACATATACTAAAATTGGAAC
U6_R	IDT	GGAACTCGAGTTTGCGTGTCATCCTTGCGC
7SL_F	IDT	CAAAACTCCCGTGCTGATCA
7SL_R	IDT	GGCTGGAGTGCAGTGGCTAT
MALAT1_F	IDT	GTCATAACCAGCCTGGCAGT
MALAT1_R	IDT	GCTTATTCCCCAATGGAGGT
SHFL_F	IDT	GGCTCTGATGAGGAAATTCGGC
SHFL_R	IDT	TCCTGGGCATCCAGATCGTTAC
matGAPDH_F	IDT	AGCCTCAAGATCATCAGCAATG
matGAPDH_R	IDT	ATGGACTGTGGTCATGAGTCCTT
RPS13_F	IDT	TCGGCTTTACCCTATCGACGCAG
RPS13_R	IDT	ACGTACTTGTGCAACACCATGTGA
ECE1_F	IDT	CGGAGCACGCGAGCTATGA
ECE1_R	IDT	CCGCCAGAAGTACCACCAAC
EPSTI1_F	IDT	GACAGAAGTGCCTGTCAAAGTG
EPSTI1_R	IDT	GCCGTTTCAGTTCCAGTAATTC
IPO7_F	IDT	TCAATTTTGGAGGCCCAGCA
IPO7_R	IDT	AGGTTGCATGATCAGGTCCC
MAN1A2_F	IDT	CACATGATGATGTACAGCAGAGC
MAN1A2_R	IDT	ATCGAACAGCAGGATTACCTGA
RELL1_F	IDT	GCAGTGGCACAGAGTAGCAG
RELL1_R	IDT	CAGTGCAGCCTTACCAGTTG
TNPO3_F	IDT	ACATTGCAGCTCGTGTACCA
TNPO3_R	IDT	AGCATGACTCCACATCCTGC
circECE1_F	IDT	ACCTCTGGGAACACAACCAA
circECE1_R	IDT	GCCGTTGGGGTATGCGTC
circEPSTI1_F	IDT	AAGCTGAAGAAGCTGAACTC
circEPSTI1_R	IDT	GTGTATGCACTTGTGTATTGC
circIPO7_F	IDT	GGGCCAGATGAAGAAGGTAGT
circIPO7_R	IDT	GAGCCTGCATTACAGGTCTGAT
circMAN1A2_F	IDT	TAGGACATATGGGTGGGGAC
circMAN1A2_R	IDT	TGCTTCTTCCAAGGCCTTCT
circRELL1_F	IDT	ATGTCTGTTAGTGGGGCTGA
circRELL1_R	IDT	TATCTGCTACCATCGCCTTT
circTNPO3_F	IDT	GCTAATCGGCGCACAGAAAT
circTNPO3_R	IDT	GGTCTGAGATCTCCCATGCA
CpG DNA	IDT, RNase Free HPLC Purification, * Phosphorothioate Bond	T*C*G*T*C*G*T*T*T*T*G*T*C*G*T*T*T*T*G*T*C*G*T*T
siCircRELL1	Horizon Life Sciences	AGUAGCAGCGAAUGCUGAUGUUU
**Chemicals, enzymes and other reagents**		
JQ1	Cayman Chemical	CAS: 1268524-70-4
Human IFN-β	Peprotech	300-02BC
Human IFN-γ	Peprotech	300-02
Transporter 5	Polysciences	26008
CD90.1 MicroBeads	Miltenyi	130-121-273
Direct-zol RNA miniprep kit	Zymo	R2053
ReverTra Ace qPCR RT master mix	Toyobo	FSQ-101
Thunderbird Next SYBR qPCR mix	Toyobo	QPX-201
TruSeq Stranded Total RNA Ribo-Zero Gold	Illumina	RS-122-2303
Stranded Total RNA Prep with Ribo-Zero Plus	Illumina	20040525
ERCC spike-in controls	ThermoFisher	4456740
**Software**		
Cutadapt	M. Martin 2011 *EMBnet.journal*	N/A
STAR	Dobin et al, 2012 *Bioinformatics*	N/A
CIRCExplorer3 (CLEAR)	Ma et al, 2019 *Genomics Proteomics Bioinformatics*	N/A
RankProd R package	Del Carratore et al, 2017 *Bioinformatics*	N/A
FIMO version 5.5.4	Grant et al, 2011 *Bioinformatics*	N/A
FlowJo v.10.9.0	BD	N/A
miEAA v.2.0	Kern et al, 2020*Nucleic Acids Research*	N/A
Cytoscape 3.10	Shannon et al, 2003*Genome Research*	N/A
DIANA-TarBase v8.0	Karagkouni et al, 2018*Nucleic Acids Research*	N/A
WebGestalt	Liao et al, 2019*Nucleic Acids Research*	N/A
Ingenuity Pathway Analysis v.1.22.01	Qiagen	N/A
**Other**		
Illumina NextSeq 550	Illumina	N/A
Illumina NovaSeq SP	Illumina	N/A
CytoFlex S	Beckman Coulter	N/A
StepOnePlus real-time PCR system	ThermoFisher	N/A

### Methods and protocols

#### Biosafety

All experiments using viruses were performed at the National Institutes of Health (NIH) in Bethesda, Maryland, in Biosafety Level (BSL) 2+ facilities. The BSL-2+ laboratory is equipped with facilities ensuring enhanced safety, such as biological safety cabinets, personal protective equipment, and protocols for handling infectious materials. Research protocols were reviewed and received approval from the Division of Occupational Health and Safety, Office of Research Services at NIH, in accordance with the NIH Guidelines for Research and the Department of Health and Human Services (USA).

#### Cells and viruses

Vero (ATCC #CCL-81) were maintained in Dulbecco’s modified eagle medium (DMEM, Gibco #11965-092) supplemented with 5% fetal bovine serum (FBS, Gibco #16000044), 1 mM sodium pyruvate (Gibco # 11360070), 2 mM L-glutamine (Gibco # A2916801), 100 U/mL penicillin-streptomycin (Gibco # 15070063). MRC-5 (ATCC #CCL-171) were maintained in DMEM (Gibco) supplemented with 10% FBS, 1 mM sodium pyruvate, 2 mM L-glutamine, 100 U/mL penicillin-streptomycin. NIH 3T3 (ATCC #CRL-1658) and NIH 3T12 (ATCC #CCL-164) were maintained in DMEM (Corning #10-017-CV) supplemented with 8% FBS, 2 mM L-glutamine, 100 U/mL penicillin-streptomycin. A20 HE-RIT B cells harbor a recombinant MHV68 expressing a hygromycin-eGFP cassette (Forrest and Speck, 2008) and doxycycline-inducible RTA (Santana et al, 2017). A20 HE-RIT were maintained in RPMI supplemented with 10% FBS, 100 U/mL penicillin-streptomycin, 2 mM L-glutamine, 50 µM beta-mercaptoethanol, 300 µg/ml hygromycin B, 300 µg/mL gentamicin, and 2 µg/mL puromycin. iSLK-BAC16 (Brulois et al, 2012) were maintained in DMEM (Gibco #11965-092) supplemented with 10% Tet system-approved FBS (Takara #631368), 50 µg/mL hygromycin B, 0.1 mg/mL gentamicin, 1 µg/mL puromycin, 100 U/mL penicillin-streptomycin. 293T (ATCC #CRL-3216) cells were maintained in DMEM supplemented with 10% FBS and 100 U/mL penicillin-streptomycin. Lymphoma cell lines, EBV-positive and negative Akata (designated with (+) or (-)) (Komano et al, 1998), Daudi (ATCC #CCL-213), and BJAB (DSMZ #ACC757) were maintained in Roswell Park Memorial Institute (RPMI) 1640 (Gibco #11875093) supplemented with 10% FBS and 100U/ml penicillin-streptomycin. HDLEC (PromoCell #C-12216) were maintained in EBM-2 basal medium (Lonza #CC-3156) supplemented with EGM-2 SingleQuots supplements (Lonza #CC-4176). Cell culture models were periodically subjected to mycoplasma testing by PCR or enzymatic reporter assays and found negative.

#### Virus stock preparation and titration

**HSV-1**. Vero cells were infected with KOS (Smith, 1964) or strain 17 at a low multiplicity of infection (MOI; ~0.01 plaque forming units (PFU)/cell) and harvested when cells were sloughing from the sides of the vessel. Supernatant and cell fraction were collected and centrifuged at 4000 × *g* 4 °C for 10 min. The subsequent supernatant fraction was reserved. The pellet fraction was freeze (−80 °C 20 min)/thawed (37 °C 5 min) for three cycles, sonicated for 1 min, and centrifuged at 2000 × *g* 4 °C for 10 min. The final virus stock was composed of the cell-associated virus and reserved supernatant virus fractions. Viral stocks were titered by plaque assay on Vero cells.

**KSHV**. iSLK-BAC16 cells were induced with 1 µg/mL doxycycline and 1 mM sodium butyrate for 3 days. Cell debris was removed from the supernatant fraction by centrifuging at 2000 × *g* 4 °C for 10 min and filtering with a 0.45 polyethersulfone membrane. Virus was concentrated after a 16,000 × *g* 4 °C 24 h spin and resuspended in a low volume of DMEM media (~1000-fold concentration). To assess viral infectivity, LECs were infected with serial dilutions of BAC16 stock and assessed using CytoFlex S (Beckman Coulter) for GFP+ cells at 3 days post infection. BAC16 contains a constitutively expressed GFP gene within the viral genome. Based on these assays, BAC16 stock was used at a 1:60 dilution, resulting in 70% infection for LEC (MOI 1).

**MHV68**. NIH 3T12-based cell lines were infected at a low MOI with MHV68 until 50% cytopathic effect was observed. Infected cells and conditioned media were dounce homogenized and clarified at 600 × *g* 4 °C for 10 minutes. Clarified supernatant was further centrifuged at 3000 × *g* 4 °C for 15 min and then 10,000 × *g* 4 °C for 2 h to concentrate 40-fold in DMEM. H2B-YFP was prepared and titered using plaque assays in NIH 3T12 cells.

#### De novo infection

**HSV-1**. Confluent MRC-5 cells were infected with 0.1 or 10 PFU per cell. Virus was adsorbed in PBS for 1 h at room temperature. Viral inoculum was removed, and cells were washed quickly with PBS before adding on DMEM media supplemented with 2% FBS. Zero-hour time point was considered after adsorption of infected monolayers when cells were place at 37 °C.

**KSHV**. Subconfluent LEC were infected with BAC16 at an approximate MOI of 1 (70% cells infected), as assessed by GFP+ cells at 3 dpi. Virus was adsorbed in a low volume of media containing 8 µg/mL polybrene for 8 h at 37 °C, after which viral inoculum was removed and replaced with fresh media. Zero-hour time point was when virus was added and cells were first placed at 37 °C.

**MHV68**. Subconfluent NIH3T3 fibroblasts were infected with 5 PFU per cell. Virus was adsorbed in a low volume of DMEM media supplemented with 8% FBS for 1 h at 37 °C, prior to overlay with fresh media. Zero-hour time point was when virus was first added and cells were placed at 37 °C.

#### HSV-1 mouse infections

Female 8-week-old BALB/cAnNTac (Taconic Biosciences) mice were infected with 10^5^ PFU HSV-1 (strain 17) via the ocular route. The appropriate number of animals for a given experiment were purchased and maintained in microisolator cages at a density of 5 mice per cage. Sample size calculation was not performed. Mice were randomized prior to infection with HSV-1 and were subsequently randomly sampled for tissue harvest. Latently infected trigeminal ganglia were harvested approximately four weeks after primary infection and immediately processed. For explant-induced reactivation, latently infected trigeminal ganglia were explanted into culture (DMEM/1% FBS) for 12 h at 37 °C/5% CO_2_ in the presence of vehicle (DMSO) or 2 μM JQ1+ to enhance reactivation (Cayman Chemical CAS: 1268524-70-4). Isolated sensory ganglia were pooled and randomized into groups of 6 prior to RNA isolation. Pooled ganglia were homogenized in 1 ml TriPure isolation reagent (Roche) using lysing matrix D on a FastPrep24 instrument (3 cycles of 40 seconds at 6 m/s). 0.2 ml chloroform was added for phase separation using phase lock gel heavy tubes and RNA isolation from the aqueous phase was obtained by using ISOLATE II RNA Mini Kit (Bioline). All animal care and handling were done in accordance with the U.S. National Institutes of Health Animal Care and Use Guidelines and as approved by the National Institute of Allergy and Infectious Diseases Animal Care and Use Committee (Protocol LVD40E, T.M.K.). Samples were identified during RNA-Seq analysis (no blinding).

#### Lytic reactivation

**KSHV**. Subconfluent monolayers of iSLK-BAC16 were induced with 1 µg/mL doxycycline, 1 mM sodium butyrate in DMEM media supplemented with 2% Tet system-approved FBS. Zero-hour time point was when induction media was added and cells were first placed at 37 °C.

**MHV68**. One day prior to induction, A20 HE-RIT cells were subcultured at a 1:3 dilution in media lacking antibiotics. Cells were seeded subconfluently and induced for 24 h with RPMI media containing 5 µg/ml doxycycline and 20 ng/ml 12-O-tetradecanoylphorbol-13-acetate (TPA).

#### rRNA-depleted total RNA-Seq

Total RNA was isolated from cells using the Direct-zol RNA MiniPrep kit (Zymo Research R2053), following manufacturer’s instructions. ERCC spike-in controls (ThermoFisher 4456740) were added to 500–1000 ng of total RNA. RNA was sent to the NCI CCR-Illumina Sequencing facility for library preparation and sequencing. RNA was rRNA depleted and directional cDNA libraries were generated using either Stranded Total RNA Prep with Ribo-Zero Plus (Illumina # 20040525) or TruSeq Stranded Total RNA Ribo-Zero Gold (Illumina #RS-122-2303). 2–4 biological replicates were sequenced for all samples. Sequencing was performed at the National Cancer Institute Center for Cancer Research Frederick Sequencing Facility using the Illumina NextSeq 550 or Illumina NovaSeq SP platform to generate 150 bp paired-end reads.

#### RNA extraction and RT-qPCR

Total RNA was extracted with Direct-zol RNA miniprep kit with on-column DNase I digestion (Zymo Research #R2053). 0.5 to 1 μg of total RNA was used for reverse-transcription with ReverTra Ace qPCR RT master mix (Toyobo #FSQ-101) and quantitative PCR (qPCR) was performed with Thunderbird Next SYBR qPCR mix (Toyobo #QPX-201) and StepOnePlus real-time PCR system (ThermoFisher) following manufacturer’s instructions.

#### Measuring viral genomes

The cell fraction was isolated from infection models. Cell pellets were washed with 1x PBS and lysed using 0.5% SDS, 400 µg/mL proteinase K, 100 mM NaCl. Samples were incubated at 37 °C for 12–18 h and heat inactivated for 30 minutes at 65 °C. DNA samples were serial diluted 1:1000 and measured using qPCR with primers specific to HSV-1 UL23 and human *GAPDH*. Standard curves were generated using purified genomic stocks (HSV-1 bacterial artificial chromosome and human genome Promega #G1471). Absolute copy number of genomic stocks was determined using droplet digital PCR (Biorad QX600). Values were plotted as follows: ${{{\rm{viral}}}}\;{{{\rm{genomes}}}}/{{{\rm{cell}}}} = \frac{{{{{\rm{viral}}}}\;{{{\rm{gene}}}}\;{{{\rm{copy}}}}\;{{{\rm{number}}}}}}{{{{{\rm{host}}}}\;{{{\rm{gene}}}}\;{{{\rm{copy}}}}\;{{{\rm{number}}}}/2}}$.

#### Viral nuclease ectopic expression

3 × 10^6^ 293T cells were seeded to 10 cm petri dishes and incubated overnight. Cells were transfected with 8 µg of plasmid DNAs (pCMV-Thy1.1-F2A-dsGFP/muSOX/SOX/vhs/BGLF5) using 48 μl Transporter 5 (Polysciences #26008) and 1 ml Opti-MemI (Gibco #31985070). After 24 h, cells were resuspended in 5 ml staining buffer [1x PBS Gibco # 10010023 supplemented with 2 mM ethylenediaminetetraacetic acid (Sigma) and 0.5% FBS (Gibco)] and mouse Thy1.1-expressing cells were magnetically enriched with CD90.1 MicroBeads (Miltenyi #130-121-273). Cells were stained with Alexa Fluor 647 anti-mouse Thy-1.1 Antibody (BioLegend; clone OX-7) and enrichment was confirmed with CytoFlex S (Beckman Coulter). 70–80% of cells were positive for Thy1.1 after sorting and lysed with TRI reagent (Zymo Research #R2050-1) for RT-qPCR.

#### Immune stimulation of cell culture models

Confluent monolayers of MRC-5 and LEC were treated with a variety of immunostimulants. 10 µg/mL lipopolysaccharide (Millipore Sigma L3024), 4 µg/mL CpG DNA (IDT), 10 µg/mL poly I:C (Millipore Sigma #P1530), or 25 ng/mL recombinant human IFN-β and γ (Peprotech #300-02BC and #300-02) was added to culture media. After the indicated treatment time (8, 24, 48, or 72 h), RNA was isolated from the cell fraction. For Akata-, Daudi, or BJAB, 10 µg/mL lipopolysaccharide, 10 µg/mL poly I:C, 10 ng/mL IFN-β, or 500 ng/mL IFN-γ were added to culture media. After 24 h RNA was isolated from the cell fraction.

#### Interferon stimulation

Confluent monolayers of MRC-5 and LEC were treated with 25 ng/mL recombinant human IFN-β and γ (Peprotech #300-02BC and #300-02) in the culture media. For Akata(-) 10 ng/mL IFN-β was added to culture media. After 48 h RNA was isolated from the cell fraction. Human peripheral blood mononuclear cells (PBMCs) were prepared from buffy coats. Buffy coats from two different donors were purified with Ficoll-Paque PLUS (GE Healthcare #17144003). PBMCs were treated with red blood cell lysis buffer (BioLegend #420301) and washed three times with phosphate-buffered saline (Gibco #10010023). 10 or 30 ng/mL recombinant human IFN-β (Peprotech #300-02BC) was added to the culture media. After 24 h RNA was isolated from the cell fraction.

#### Immunostaining and flow cytometry

Cells were washed with PBS, detached with Accutase (BioLegend), and mixed with antibodies in staining buffer (PBS, 0.5% FBS, 2 mM EDTA) for 20 min at room temperature. Alexa Fluor 647 anti-mouse Thy-1.1 Antibody (BioLegend; clone OX-7) was used. Fluorescence was measured by CytoFlex S (Beckman Coulter) flow cytometer and analyzed with FlowJo 10.9.0 (BD). Doublets were removed and Allophycocyanin (APC) channel was used to quantify Thy1.1 levels at cell surface.

#### In silico circRNA-miRNA-mRNA network predictions

Four commonly-regulated human circRNAs (hsa_circ_0001730, hsa_circ_0006990, hsa_circ_0006646, hsa_circ_0001769) were examined for miRNA-binding sites using CircInteractome (Dudekula et al, 2016). Experimentally supported miRNA-mRNA interactions were then added to the network based on DIANA-TarBase v8.0 (Karagkouni et al, 2018). Cytoscape 3.10 was used for visualizing the network (Shannon et al, 2003). Overrepresentation analysis of predicted target miRNAs was performed with miEAA 2.0 (Kern et al, 2020). Enriched Gene Ontology terms (http://geneontology.org), *p*-values, and miRNAs that were predicted to be regulated by circRNAs are shown.

#### Overrepresentation analysis (ORA)

ORA was performed using a list of genes colinear to circRNAs detected in a given model (>10 raw BSJ read counts). ORA was performed using WebGestalt (Liao et al, 2019) and plotted using SRplot (https://www.bioinformatics.com.cn/en).

#### Ingenuity pathway analysis (IPA)

IPA (Qiagen) was performed on de novo lytic infection RNA-Seq datasets for HSV-1 (MRC-5 infected at MOI (multiplicity of infection) of 10 for 12 h, 4 biological replicates), HCMV (MRC-5 infected at MOI of 3 for 72 h, 2 biological replicates), and KSHV (LEC infected at MOI of 1 for 72 h, 2 biological replicates). Log_2_ fold change (log_2_FC, Infected/Uninfected) was calculated from ERCC normalized data. Only genes with an average log_2_FC > 1 were used for IPA. Bubble plots were generated using SRplot (https://www.bioinformatics.com.cn/en).

#### circRELL1 functional studies

**Loss of function**. Subconfluent MRC-5 were incubated for 8 h at 37 °C in OptiMEM (ThermoFisher #31985062) with 20 nM siRNA and Lipofectamine RNAiMAX Transfection Reagent (ThermoFisher #13778150). siRNAs were ON-TARGETplus Non-targeting Control Pool (Horizon #D-001810-10-05) and ON-TARGETplus siCircRELL1, previously published as “siCirc1400-2” (Tagawa et al, 2018). This siRNA target back-splice junctions of circRELL1 (circ_0001400) and does not affect the linear counterpart RNA, *RELL1*. After 8 h transfection media was removed and cells incubated in 1x DMEM supplemented with 2% FBS, 1 mM sodium pyruvate, 2 mM L-glutamine for an additional 40 h at 37 °C. If indicated, cells were infected with HSV-1 strain KOS at an MOI of 0.1 or 10 PFU/cell and collected at 12 h post infection.

**Gain of function**. Lentiviral vectors expressing circGFP or circRELL1 with a fluorescent (mCherry) reporter were cloned and pseudotyped with VSV-G were cloned and purified by VectorBuilder (Tagawa et al, 2023). The circRELL1 insert contains exons 4–6 of the colinear transcript (NCBI RefSeq NM_001085400.2). Inserts are flanked by *ZKSCAN1* intronic sequences which facilitate circularization of the internal sequence (Kramer et al, 2015). Subconfluent MRC-5 were incubated with lentiviral vectors in 1x DMEM supplemented with 10% FBS, 1 mM sodium pyruvate, 2 mM L-glutamine, 8 µg/mL polybrene. Cells were incubated with lentivirus at 37 °C for 18 h, before media was replaced. MRC-5 were infected with comparable infectious doses of circGFP and circRELL1 lentivirus, such that 70% of cell were mCherry+ at three days post infection. Forty hours after lentivirus infection, MRC-5 were infected with HSV-1 strain KOS at an MOI of 10 PFU/mL and the cell fraction collected at 12 h post HSV-1 infection.

#### Gene quantitation

RNA-Sequencing reads were trimmed using Cutadapt (Martin, 2011) and the following parameters: --pair-filter=any, --nextseq-trim=2, --trim-n, -n 5, --max-n 0.5, -0 5, -q 20, -m 15. Trimmed reads were mapped using STAR (Dobin et al, 2012) with 2-pass mapping to concatenated genome assemblies which contain the host genome (hg38 or mm39) + virus genome (KT899744.1, NC_001806.2, NC_006273.2, NC_009333.1, MH636806.1) + ERCC spike-in controls. Details on mapping assemblies are included below. RNA STAR mapping parameters are as follows: --outSJfilterOverhangMin 15 15 15 15, --outFilterType BySJout, --outFilterMultimapNmax 20, --outFilterScoreMin 1, --outFilterMatchNmin 1, --outFilterMismatchNmax 2, --outFilterMismatchNoverLmax 0.3, --outFilterIntronMotifs None, --alignIntronMin 20, --alignIntronMax 2000000, --alignMatesGapMax 2000000, --alignTranscriptsPerReadNmax 20000, --alignSJoverhangMin 15, --alignSJDBoverhangMin 15, --alignEndsProtrude 10 ConcordantPair, --chimSegmentMin 15, --chimScoreMin 15, --chimScoreJunctionNonGTAG 0 --chimJunctionOverhangMin 18, --chimMultimapNmax 10. RNA STAR GeneCount (per gene read counts) files were used for transcript quantitation. The only RNA-Seq data not generated in-house is from HCMV infection (Data ref: Oberstein and Shenk, 2017) (Table 3). This data did not contain ERCC spike-in controls and thus was normalized as Transcripts per Million (TPM). For all other models, ERCC reads were used to generate standard curves similar to (Schertzer et al, 2020), using their known relative concentrations. All biological replicates had ERCC-derived standard curves with R^2^ > 0.9. ERCC normalized gene counts were calculated as follows:

$${{{{{\rm{Log}}}}}}_2{{{{{\rm{RPKM}}}}}}{:}\,{{{\rm{Log}}}}_2\left( {\frac{{{{{\rm{Raw}}}}\;{{{\rm{gene}}}}\;{{{\rm{counts}}}}}}{{{{{\rm{gene}}}}\;{{{\rm{size}}}}\;{{{\rm{in}}}}\;kb {\times} {{{\rm{Million}}}}\;{{{\rm{total}}}}\;{{{\rm{reads}}}}}}} \right)$$

*Raw gene counts includes forward spliced reads and excludes reads containing BSJ.

$${{{{{\rm{ERCC}}}}}}\;{{{{{\rm{norm}}}}}}.{{{{{\rm{gene}}}}}}\;{{{{{\rm{counts}}}}}}{:}\left( {2^{\left( {\frac{{{{{\rm{Log}}}}_2\left( {{{{\rm{RPKM}}}}} \right) - {{{\rm{ERCC}}}}\;{{{\rm{derived}}}}\;{{{\rm{intercept}}}} \, \left( b \right)}}{{{{{\rm{ERCC}}}}\;{{{\rm{derived}}}}\;{{{\rm{slope}}}} \, \left( y \right)}}} \right)}} \right)/10,000$$

**Table 3:** RNA-Seq data analyzed in this study.

Model			Metadata	Accession
HSV-1	Lytic infection	MRC-5 cells infected with HSV-1 strain KOS (MOI 10 PFU/cell) for 12 or 24 h. PMID: 36724259	*n* = 2–4 Illumina 150 PE	SRR19779319, SRR19779318, SRR19787559
	Latent infection	Female 8-week-old BALB/cAnTAC mice infected with HSV-1 strain 17 (10^5^ PFU) via the ocular route. Latently infected trigeminal ganglion (TG) were harvested 4 weeks after primary infection.	*n* = 4 Illumina 150 PE	SRR19792335, SRR19792334
	Lytic reactivation	Female 8-week-old BALB/cAnTAC mice infected with HSV-1 strain 17 (10^5^ PFU) via the ocular route. Trigeminal ganglia were explanted (TG explant) ~4 weeks after primary infection in the presence of vehicle or 2 µM JQ1 for 12 h.	*n* = 2–3 Illumina 150 PE	SRR25824398, SRR25824397, SRR25824394, SRR25824396
HCMV-Lytic infection		MRC-5 cells infected with HCMV strain TB40/E (MOI 3 PFU/cell) for 24 or 72 h. PMID: 28874566	*n* = 2 Illumina 100 PE	SRR5629593, SRR5629594, SRR5629591, SRR5629592, SRR5629589, SRR5629590, SRR5629587, SRR5629588, SRR5629577, SRR5629578, SRR5629575, SRR5629576, SRR5629573, SRR5629574, SRR5629571, SRR5629572
KSHV	Lytic infection	LEC infected with KSHV strain BAC16 (MOI 1 PFU/cell) for 24 or 72 h. PMID: 36724259	*n* = 2 Illumina 150 PE	SRR20020769, SRR25816558, SRR20020770
	Lytic reactivation	iSLK-BAC16 induced with 1 µg/mL Doxycycline 1 mM Sodium Butyrate for 24 or 72 h. PMID: 36724259	*n* = 4 Illumina 150 PE	SRR25816557, SRR20020761, SRR25816556, SRR20020757, SRR20020758
MHV68	Lytic infection	NIH 3T3 cells infected with MHV68 strain H2B-YFP (MOI 5 PFU/cell) for 6 or 18 h.	*n* = 2 Illumina 150 PE	SRR19792326, SRR25823339, SRR19792325
	Lytic reactivation	A20 HE-RIT cells induced with 5 µg/ml Dox and 20 ng/ml TPA for 6 or 24 h.	*n* = 2 Illumina 150 PE	SRR19792324, SRR25823338, SRR19792321
Interferon treatment		MRC-5, LEC, or Akata- cells treated with IFN-β or -γ or- for 48 h.	*n* = 3 Illumina 150 PE	SRR25905055, SRR25905049, SRR25905048, SRR25905050, SRR25905054, SRR25905051, SRR25905053, SRR25905052
EBV-Latent infection		Akata+ (EBV positive) or Akata- (EBV negative) cells. 074301 Arraystar Human CircRNA microarray V2. PMID: 36724259	*n* = 3 Microarray	GSE206824

Overview of bulk RNA-Seq data analyzed in this study. If data from any of the sample sets was previously published we have included PubMed reference numbers.

#### CircRNA quantitation

RNA-Seq data was trimmed (Cutadapt) and aligned (STAR, 2-pass) as described in the gene quantitation section above. Note that we required a minimum of 18 nucleotides flanking any chimeric BSJ calls to ensure high-confidence in circRNA quantitation. Back-splice junctions (BSJ) were quantified using CIRCExplorer3 (CLEAR) pipeline (Ma et al, 2019) and normalized as TPM (HCMV data) or relative to ERCC spike in controls (all other data). BSJ variants are reported relative to their colinear gene products and circbase annotations (http://www.circbase.org/) (Glažar et al, 2014). Gene length for circRNA was treated as 0.15 kb as that is the total read length and full circRNA size is unknown. ERCC normalized circRNA counts were calculated as above.

#### Differentially expressed circRNA (DEC) calling

**Bulk RNA-Seq data.** Up- or down-regulated circRNAs had a raw BSJ count across the sample set >10, log_2_FC > 0.5, and *p*-value < 0.05. With the exception of interferon-stimulated RNA-Seq data (Fig. 4), significance was calculated by rank product paired-analysis with RankProd R package (Del Carratore et al, 2017). For interferon-stimulated RNA-Seq data EdgeR was used to calculate statistical significance.

**EBV microarray data.** Data was previously published in (Data ref: Tagawa et al, 2023), comparing Akata(+) and Akata(-) cells assessed by microarray (074301 Arraystar Human CircRNA microarray V2). Upregulated circRNAs had a log_2_FC > 0.5 and *p*-value < 0.05. Significance was calculated by rank product paired-analysis with RankProd R package (Del Carratore et al, 2017).

#### Analysis of SOX substrate-targeting motif

CircRNA subsets were binned using the average log_2_ fold change (iSLK-BAC16 3 days post induction/uninduced) from four biological replicates. CircRNA back splice junctions were cross-referenced to circAtlas (Wu et al, 2020) to determine expected full-length sequences. Motif scanning was performed using FIMO (Grant et al, 2011) version 5.5.4 looking for exact matches to “UGAAG” with the following run parameters: --verbosity 1 --bgfile --nrdb-- --thresh 0.01 --no-pgc.

#### Transcript isoform analysis

RNA-Sequencing reads were trimmed using Cutadapt (Martin, 2011) and the following parameters: --pair-filter=any, --nextseq-trim=2, --trim-n, -n 5, --max-n 0.5, -0 5, -q 20, -m 15. Trimmed reads were mapped and quantified using Salmon quant Version 1.10.1 (Patro et al, 2017) using a transcript fasta reference and the following parameters: --kmerLen 31, --incompatPrior 0.0, --biasSpeedSamp 5, --fldMax 1000, --fldMean 250, --fldSD 25, --forgettingFactor 0.65, --maxReadOcc 100, --numBiasSamples 2000000, --numAuxModelSamples 5000000, --numPreAuxModelSamples 5000, --numGibbsSamples 0, --numBootstraps 0, --thinningFactor 16, --sigDigits 3, --vbPrior 1e-05. The reference transcriptome fasta was created using gffread v0.12.7 (Pertea and Pertea, 2020) with the following parameters: -w, -g (reference genome: GRCh38 version 36, Ensembl 102). Salmon transcript and gene quantitation files were normalized to ERCC spike-in reads as detailed in the main Methods section.

#### Genome assemblies

HSV-1, strain KOS: KT899744.1, with the corresponding coding sequence (CDS) annotation used for transcript quantification.

HSV-1, strain 17: NC_001806.2, with the corresponding CDS annotation used for transcript quantification.

HCMV: NC_006273.2, with the corresponding CDS annotation used for transcript quantification.

KSHV: NC_009333.1, with the corresponding CDS annotation used for transcript quantification.

MHV68: MH636806.1 (O’Grady et al, 2016) modified to remove the beta-lactamase gene (Δ103,908-105,091), with the corresponding CDS annotation used for transcript quantification.

Human: hg38, gencode.v36.

Mouse: mm39, gencode.vM29.

ERCC spike-in: available from ThermoFisher (#4456740).

## Supplementary information

Appendix

Dataset EV1

Dataset EV2

Dataset EV3

Dataset EV4

Dataset EV5

Dataset EV6

Dataset EV7

Source Data Fig. 3

Source Data Fig. 5

58459 Peer Review File

Expanded View Figures

## Data Availability

Additional information about data analyzed in this study is present in Table 3. HSV-1 infection RNA-Seq data: Sequence Read Archive (SRA) database SRR19779319, SRR19779318, SRR19787559 https://www.ncbi.nlm.nih.gov/sra?term=SRP383035. https://www.ncbi.nlm.nih.gov/sra?term=SRP383161. HSV-1 murine infection RNA-Seq data: SRA database SRR19792335, SRR19792334, SRR25824398, SRR25824397, SRR25824394, SRR25824396 https://www.ncbi.nlm.nih.gov/sra?term=SRP383241. KSHV infection RNA-Seq data: SRA database SRR20020769, SRR20020770, SRR20020761, SRR20020757, SRR20020758, SRR25816558, SRR25816557, SRR25816556 https://www.ncbi.nlm.nih.gov/sra?term=SRP385335. MHV68 infection RNA-Seq data: SRA database SRR19792326, SRR19792325, SRR19792324, SRR19792321, SRR25823338, SRR25823339 https://www.ncbi.nlm.nih.gov/sra?term=SRP383239. Interferon stimulation RNA-Seq data: SRA database SRR25905055, SRR25905049, SRR25905048, SRR25905050, SRR25905054, SRR25905051, SRR25905053, SRR25905052. https://www.ncbi.nlm.nih.gov/sra?term=SRP458394.

## References

[CR1] Abere B, Zhou H, Li J, Cao S, Toptan T, Grundhoff A, Fischer N, Moore PS, Chang Y, Racaniello VR (2020) Merkel cell polyomavirus encodes circular RNAs (circRNAs) enabling a dynamic circRNA/microRNA/mRNA regulatory network. mBio 11:e03059–03020 33323517 10.1128/mBio.03059-20 PMC7773998

[CR2] Abernathy E, Clyde K, Yeasmin R, Krug LT, Burlingame A, Coscoy L, Glaunsinger B (2014) Gammaherpesviral gene expression and virion composition are broadly controlled by accelerated mRNA degradation. PLOS Pathog 10:e1003882 24453974 10.1371/journal.ppat.1003882 PMC3894220

[CR3] Abernathy E, Glaunsinger B (2015) Emerging roles for RNA degradation in viral replication and antiviral defense. Virology 479-480:600–608 25721579 10.1016/j.virol.2015.02.007 PMC4424162

[CR4] Ablashi DV, Berneman ZN, Kramarsky B, Whitman J, Asano Y, Pearson GR (1995) Human herpesvirus-7 (HHV-7): current status. Clin Diagn Virol 4:1–13 15566823 10.1016/0928-0197(95)00005-s

[CR5] Abrisch RG, Eidem TM, Yakovchuk P, Kugel JF, Goodrich JA (2015) Infection by herpes simplex virus 1 causes near-complete loss of RNA polymerase II occupancy on the host cell genome. J Virol 90:2503–2513 26676778 10.1128/JVI.02665-15 PMC4810688

[CR6] Ashwal-Fluss R, Meyer M, Pamudurti Nagarjuna R, Ivanov A, Bartok O, Hanan M, Evantal N, Memczak S, Rajewsky N, Kadener S (2014) circRNA biogenesis competes with Pre-mRNA splicing. Mol Cell 56:55–66 25242144 10.1016/j.molcel.2014.08.019

[CR7] Baillargeon J, Piper J, Leach CT (2000) Epidemiology of human herpesvirus 6 (HHV-6) infection in pregnant and nonpregnant women. J Clin Virol 16:149–157 10738135 10.1016/s1386-6532(99)00086-4

[CR8] Bate SL, Dollard SC, Cannon MJ (2010) Cytomegalovirus seroprevalence in the United States: the national health and nutrition examination surveys, 1988–2004. Clin Infect Dis 50:1439–1447 20426575 10.1086/652438 PMC11000537

[CR9] Birkenheuer CH, Danko CG, Baines JD (2018) Herpes simplex virus 1 dramatically alters loading and positioning of RNA polymerase II on host genes early in infection. J Virol 92:e02184–17 29437966 10.1128/JVI.02184-17 PMC5874419

[CR10] Bradley H, Markowitz LE, Gibson T, McQuillan GM (2013) Seroprevalence of herpes simplex virus types 1 and 2—United States, 1999–2010. J Infect Dis 209:325–333 24136792 10.1093/infdis/jit458

[CR11] Brulois KF, Chang H, Lee AS-Y, Ensser A, Wong L-Y, Toth Z, Lee SH, Lee H-R, Myoung J, Ganem D et al (2012) Construction and manipulation of a new Kaposi’s sarcoma-associated herpesvirus bacterial artificial chromosome clone. J Virol 86:9708–9720 22740391 10.1128/JVI.01019-12 PMC3446615

[CR12] Cesarman E, Damania B, Krown SE, Martin J, Bower M, Whitby D (2019) Kaposi sarcoma. Nat Rev Dis Primers 5:9 30705286 10.1038/s41572-019-0060-9 PMC6685213

[CR13] Chen C-y, Sarnow P (1995) Initiation of protein synthesis by the eukaryotic translational apparatus on circular RNAs. Science 268:415–417 7536344 10.1126/science.7536344

[CR14] Chen T-C, Tallo-Parra M, Cao QM, Kadener S, Böttcher R, Pérez-Vilaró G, Boonchuen P, Somboonwiwat K, Díez J, Sarnow P (2020) Host-derived circular RNAs display proviral activities in Hepatitis C virus-infected cells. PLOS Pathog 16:e1008346 32764824 10.1371/journal.ppat.1008346 PMC7437927

[CR15] Chen YG, Chen R, Ahmad S, Verma R, Kasturi SP, Amaya L, Broughton JP, Kim J, Cadena C, Pulendran B et al (2019) N6-Methyladenosine modification controls circular RNA immunity. Mol Cell 76:96–109.e109 31474572 10.1016/j.molcel.2019.07.016 PMC6778039

[CR16] Chen YG, Kim MV, Chen X, Batista PJ, Aoyama S, Wilusz JE, Iwasaki A, Chang HY (2017) Sensing self and foreign circular RNAs by intron identity. Mol Cell 67:228–238.e225 28625551 10.1016/j.molcel.2017.05.022 PMC5610545

[CR17] Conn Simon J, Pillman Katherine A, Toubia J, Conn Vanessa M, Salmanidis M, Phillips Caroline A, Roslan S, Schreiber Andreas W, Gregory Philip A, Goodall Gregory J (2015) The RNA binding protein quaking regulates formation of circRNAs. Cell 160:1125–1134 25768908 10.1016/j.cell.2015.02.014

[CR18] Covarrubias S, Gaglia MM, Kumar GR, Wong W, Jackson AO, Glaunsinger BA (2011) Coordinated destruction of cellular messages in translation complexes by the gammaherpesvirus host shutoff factor and the mammalian exonuclease Xrn1. PLOS Pathog 7:e1002339 22046136 10.1371/journal.ppat.1002339 PMC3203186

[CR19] Daly R, Khaperskyy DA, Gaglia MM (2020) Fine-tuning a blunt tool: regulation of viral host shutoff RNases. PLOS Pathog 16:e1008385 32267905 10.1371/journal.ppat.1008385 PMC7141626

[CR20] Del Carratore F, Jankevics A, Eisinga R, Heskes T, Hong F, Breitling R (2017) RankProd 2.0: a refactored bioconductor package for detecting differentially expressed features in molecular profiling datasets. Bioinformatics 33:2774–2775 28481966 10.1093/bioinformatics/btx292 PMC5860065

[CR21] Dobin A, Davis CA, Schlesinger F, Drenkow J, Zaleski C, Jha S, Batut P, Chaisson M, Gingeras TR (2012) STAR: ultrafast universal RNA-seq aligner. Bioinformatics 29:15–21 23104886 10.1093/bioinformatics/bts635 PMC3530905

[CR22] Dong S, Forrest JC, Liang X (2017) Murine gammaherpesvirus 68: a small animal model for gammaherpesvirus-associated diseases. Adv Exp Med Biol 1018:225–236 29052141 10.1007/978-981-10-5765-6_14

[CR23] Dowd JB, Palermo T, Brite J, McDade TW, Aiello A (2013) Seroprevalence of Epstein-Barr virus infection in U.S. children ages 6-19, 2003-2010. PLoS ONE 8:e64921 23717674 10.1371/journal.pone.0064921 PMC3661547

[CR24] Dremel SE, DeLuca NA (2019) Herpes simplex viral nucleoprotein creates a competitive transcriptional environment facilitating robust viral transcription and host shut off. eLife 8:e51109 31638576 10.7554/eLife.51109 PMC6805162

[CR25] Dremel SE, DeLuca NA (2019) Sequence Read Archive, PRJNA508787

[CR26] Dremel SE et al (2022a) Sequence Read Archive, PRJNA692715

[CR27] Dremel SE et al (2022b) Sequence Read Archive, PRJNA693164

[CR28] Dremel SE et al (2022c) Sequence Read Archive, PRJNA732212

[CR29] Dudekula DB, Panda AC, Grammatikakis I, De S, Abdelmohsen K, Gorospe M (2016) CircInteractome: a web tool for exploring circular RNAs and their interacting proteins and microRNAs. RNA Biol 13:34–42 26669964 10.1080/15476286.2015.1128065 PMC4829301

[CR30] Enuka Y, Lauriola M, Feldman ME, Sas-Chen A, Ulitsky I, Yarden Y (2016) Circular RNAs are long-lived and display only minimal early alterations in response to a growth factor. Nucleic Acids Res 44:1370–1383 26657629 10.1093/nar/gkv1367 PMC4756822

[CR31] Feng P, Everly Jr. DN, Read GS (2005) mRNA decay during herpes simplex virus (HSV) infections: protein-protein interactions involving the HSV virion host shutoff protein and translation factors eIF4H and eIF4A. J Virol 79:9651–9664 16014927 10.1128/JVI.79.15.9651-9664.2005 PMC1181552

[CR32] Forrest JC, Speck SH (2008) Establishment of B-cell lines latently infected with reactivation-competent murine gammaherpesvirus 68 provides evidence for viral alteration of a DNA damage-signaling cascade. J Virol 82:7688–7699 18495760 10.1128/JVI.02689-07 PMC2493333

[CR33] Friedl M-S, Djakovic L, Kluge M, Hennig T, Whisnant AW, Backes S, Dölken L, Friedel CC (2022) HSV-1 and influenza infection induce linear and circular splicing of the long NEAT1 isoform. PLoS ONE 17:e0276467 36279270 10.1371/journal.pone.0276467 PMC9591066

[CR34] Gaglia MM, Covarrubias S, Wong W, Glaunsinger BA (2012) A common strategy for host RNA degradation by divergent viruses. J Virol 86:9527–9530 22740404 10.1128/JVI.01230-12 PMC3416159

[CR35] Gaglia MM, Rycroft CH, Glaunsinger BA (2015) Transcriptome-wide cleavage site mapping on cellular mRNAs reveals features underlying sequence-specific cleavage by the viral ribonuclease SOX. PLOS Pathog 11:e1005305 26646420 10.1371/journal.ppat.1005305 PMC4672902

[CR36] Glažar P, Papavasileiou P, Rajewsky N (2014) circBase: a database for circular RNAs. RNA 20:1666–1670 25234927 10.1261/rna.043687.113 PMC4201819

[CR37] Gopinath D, Koe KH, Maharajan MK, Panda S (2023) A comprehensive overview of epidemiology pathogenesis and the management of herpes labialis. Viruses 15:225 10.3390/v15010225 PMC9867007 36680265

[CR38] Grant CE, Bailey TL, Noble WS (2011) FIMO: scanning for occurrences of a given motif. Bioinformatics 27:1017–1018 21330290 10.1093/bioinformatics/btr064 PMC3065696

[CR39] Griffiths P, Reeves M (2021) Pathogenesis of human cytomegalovirus in the immunocompromised host. Nat Rev Micro 19:759–773 10.1038/s41579-021-00582-z PMC8223196 34168328

[CR40] Harper KL, Mottram TJ, Anene CA, Foster B, Patterson MR, McDonnell E, Macdonald A, Westhead D, Whitehouse A (2022) Dysregulation of the miR-30c/DLL4 axis by circHIPK3 is essential for KSHV lytic replication. EMBO Rep 23:e54117 35239998 10.15252/embr.202154117 PMC9066072

[CR41] Huang X-Y, Huang Z-L, Xu Y-H, Zheng Q, Chen Z, Song W, Zhou J, Tang Z-Y, Huang X-Y (2017) Comprehensive circular RNA profiling reveals the regulatory role of the circRNA-100338/miR-141-3p pathway in hepatitis B-related hepatocellular carcinoma. Sci Rep 7:5428 28710406 10.1038/s41598-017-05432-8 PMC5511135

[CR42] Hutin S, Lee Y, Glaunsinger BA (2013) An RNA element in human interleukin 6 confers escape from degradation by the gammaherpesvirus SOX protein. J Virol 87:4672–4682 23408619 10.1128/JVI.00159-13 PMC3624381

[CR43] Jeck WR, Sorrentino JA, Wang K, Slevin MK, Burd CE, Liu J, Marzluff WF, Sharpless NE (2013) Circular RNAs are abundant, conserved, and associated with ALU repeats. RNA 19:141–157 23249747 10.1261/rna.035667.112 PMC3543092

[CR44] Karagkouni D, Paraskevopoulou MD, Chatzopoulos S, Vlachos IS, Tastsoglou S, Kanellos I, Papadimitriou D, Kavakiotis I, Maniou S, Skoufos G et al (2018) DIANA-TarBase v8: a decade-long collection of experimentally supported miRNA-gene interactions. Nucleic Acids Res 46:D239–d245 29156006 10.1093/nar/gkx1141 PMC5753203

[CR45] Karr BM, Read GS (1999) The virion host shutoff function of herpes simplex virus degrades the 5’ end of a target mRNA before the 3’ end. Virology 264:195–204 10544145 10.1006/viro.1999.9986

[CR46] Kern F, Fehlmann T, Solomon J, Schwed L, Grammes N, Backes C, Van Keuren-Jensen K, Craig DW, Meese E, Keller A (2020) miEAA 2.0: integrating multi-species microRNA enrichment analysis and workflow management systems. Nucleic Acids Res 48:W521–w528 32374865 10.1093/nar/gkaa309 PMC7319446

[CR47] Komano J, Sugiura M, Takada K (1998) Epstein-Barr virus contributes to the malignant phenotype and to apoptosis resistance in Burkitt’s lymphoma cell line Akata. J Virol 72:9150–9156 9765461 10.1128/jvi.72.11.9150-9156.1998 PMC110333

[CR48] Kramer MC, Liang D, Tatomer DC, Gold B, March ZM, Cherry S, Wilusz JE (2015) Combinatorial control of Drosophila circular RNA expression by intronic repeats, hnRNPs, and SR proteins. Genes Dev 29:2168–2182 26450910 10.1101/gad.270421.115 PMC4617980

[CR49] Kristensen L, Andersen M, Stagsted L, Ebbesen K, Hansen T, Kjems J (2019) The biogenesis, biology and characterization of circular RNAs. Nat Rev Genet 20:675–691 31395983 10.1038/s41576-019-0158-7

[CR50] Le Sage V, Cinti A, Amorim R, Mouland AJ (2016) Adapting the stress response: viral subversion of the mTOR signaling pathway. Viruses 8:152 27231932 10.3390/v8060152 PMC4926172

[CR51] Legnini I, Di Timoteo G, Rossi F, Morlando M, Briganti F, Sthandier O, Fatica A, Santini T, Andronache A, Wade M et al (2017) Circ-ZNF609 is a circular RNA that can be translated and functions in myogenesis. Mol Cell 66:22–37.e29 28344082 10.1016/j.molcel.2017.02.017 PMC5387670

[CR52] Li X, Liu C-X, Xue W, Zhang Y, Jiang S, Yin Q-F, Wei J, Yao R-W, Yang L, Chen L-L (2017) Coordinated circRNA biogenesis and function with NF90/NF110 in viral infection. Mol Cell 67:214–227.e217 28625552 10.1016/j.molcel.2017.05.023

[CR53] Liao Y, Wang J, Jaehnig EJ, Shi Z, Zhang B (2019) WebGestalt 2019: gene set analysis toolkit with revamped UIs and APIs. Nucleic Acids Res 47:W199–W205 31114916 10.1093/nar/gkz401 PMC6602449

[CR54] Liu C-X, Li X, Nan F, Jiang S, Gao X, Guo S-K, Xue W, Cui Y, Dong K, Ding H et al (2019) Structure and degradation of circular RNAs regulate PKR activation in innate immunity. Cell 177:865–880.e821 31031002 10.1016/j.cell.2019.03.046

[CR55] Ma XK, Wang MR, Liu CX, Dong R, Carmichael GG, Chen LL, Yang L (2019) CIRCexplorer3: a CLEAR pipeline for direct comparison of circular and linear RNA expression. Genom Proteom Bioinform 17:511–521 10.1016/j.gpb.2019.11.004 PMC7056929 31904419

[CR56] Maass PG, Glažar P, Memczak S, Dittmar G, Hollfinger I, Schreyer L, Sauer AV, Toka O, Aiuti A, Luft FC, Rajewsky N (2017) A map of human circular RNAs in clinically relevant tissues. J Mol Med 95:1179–1189 28842720 10.1007/s00109-017-1582-9 PMC5660143

[CR57] Macveigh-Fierro D, Cicerchia A, Cadorette A, Sharma V, Muller M (2022) The m6a reader YTHDC2 is essential for escape from KSHV SOX-induced RNA decay. PNAS 119:e2116662119 35177478 10.1073/pnas.2116662119 PMC8872733

[CR58] Martin M (2011) Cutadapt removes adapter sequences from high-throughput sequencing reads. EMBnetjournal 17:10–12

[CR59] McSwiggen DT, Hansen AS, Teves SS, Marie-Nelly H, Hao Y, Heckert AB, Umemoto KK, Dugast-Darzacq C, Tjian R, Darzacq X (2019) Evidence for DNA-mediated nuclear compartmentalization distinct from phase separation. eLife 8:e47098 31038454 10.7554/eLife.47098 PMC6522219

[CR60] Memczak S, Jens M, Elefsinioti A, Torti F, Krueger J, Rybak A, Maier L, Mackowiak SD, Gregersen LH, Munschauer M et al (2013) Circular RNAs are a large class of animal RNAs with regulatory potency. Nature 495:333–338 23446348 10.1038/nature11928

[CR61] Muller M, Glaunsinger BA (2017) Nuclease escape elements protect messenger RNA against cleavage by multiple viral endonucleases. PLoS Pathog 13:e1006593 28841715 10.1371/journal.ppat.1006593 PMC5589255

[CR62] O’Grady T, Wang X, Höner zu Bentrup K, Baddoo M, Concha M, Flemington EK (2016) Global transcript structure resolution of high gene density genomes through multi-platform data integration. Nucleic Acids Res 44:e145–e145 27407110 10.1093/nar/gkw629 PMC5062983

[CR63] Oberstein A, Shenk T (2017) Sequence Read Archive, PRJNA388483

[CR64] Okholm TLH, Sathe S, Park SS, Kamstrup AB, Rasmussen AM, Shankar A, Chua ZM, Fristrup N, Nielsen MM, Vang S et al (2020) Transcriptome-wide profiles of circular RNA and RNA-binding protein interactions reveal effects on circular RNA biogenesis and cancer pathway expression. Genome Med 12:112 33287884 10.1186/s13073-020-00812-8 PMC7722315

[CR65] Page HG, Read GS (2010) The virion host shutoff endonuclease (UL41) of herpes simplex virus interacts with the cellular cap-binding complex eIF4F. J Virol 84:6886–6890 20427534 10.1128/JVI.00166-10 PMC2903273

[CR66] Pamudurti NR, Bartok O, Jens M, Ashwal-Fluss R, Stottmeister C, Ruhe L, Hanan M, Wyler E, Perez-Hernandez D, Ramberger E et al (2017) Translation of CircRNAs. Mol Cell 66:9–21.e27 28344080 10.1016/j.molcel.2017.02.021 PMC5387669

[CR67] Park OH, Ha H, Lee Y, Boo SH, Kwon DH, Song HK, Kim YK (2019) Endoribonucleolytic cleavage of m(6)A-containing RNAs by RNase P/MRP complex. Mol Cell 74:494–507.e498 30930054 10.1016/j.molcel.2019.02.034

[CR68] Patro R, Duggal G, Love MI, Irizarry RA, Kingsford C (2017) Salmon provides fast and bias-aware quantification of transcript expression. Nat Methods 14:417–419 28263959 10.1038/nmeth.4197 PMC5600148

[CR69] Pertea G, Pertea M (2020) GFF utilities: GffRead and GffCompare. F1000Res 9:ISCB Comm J-304 32489650 10.12688/f1000research.23297.1 PMC7222033

[CR70] Rodriguez W, Srivastav K, Muller M (2019) C19ORF66 broadly escapes virus-induced endonuclease cleavage and restricts Kaposi’s sarcoma-associated herpesvirus. J Virol 93:e00373-19 10.1128/JVI.00373-19 PMC6613750 30944177

[CR71] Salzman J, Chen RE, Olsen MN, Wang PL, Brown PO (2013) Cell-type specific features of circular RNA expression. PLoS Genet 9:e1003777 24039610 10.1371/journal.pgen.1003777 PMC3764148

[CR72] Santana AL, Oldenburg DG, Kirillov V, Malik L, Dong Q, Sinayev R, Marcu KB, White DW, Krug LT (2017) RTA occupancy of the origin of lytic replication during murine gammaherpesvirus 68 reactivation from B cell latency. Pathogens 6:9 28212352 10.3390/pathogens6010009 PMC5371897

[CR73] Schertzer MD, Murvin MM, Calabrese JM (2020) Using RNA sequencing and spike-in RNAs to measure intracellular abundance of lncRNAs and mRNAs. Bio-protocol 10:e3772 33204768 10.21769/BioProtoc.3772 PMC7668557

[CR74] Shannon P, Markiel A, Ozier O, Baliga NS, Wang JT, Ramage D, Amin N, Schwikowski B, Ideker T (2003) Cytoscape: a software environment for integrated models of biomolecular interaction networks. Genome Res 13:2498–2504 14597658 10.1101/gr.1239303 PMC403769

[CR75] Shiflett LA, Read GS (2013) mRNA decay during herpes simplex virus (HSV) infections: mutations that affect translation of an mRNA influence the sites at which it is cleaved by the HSV virion host shutoff (Vhs) protein. J Virol 87:94–109 23077305 10.1128/JVI.01557-12 PMC3536374

[CR76] Smiley JR (2004) Herpes simplex virus virion host shutoff protein: immune evasion mediated by a viral RNase? J Virol 78:1063–1068 14722261 10.1128/JVI.78.3.1063-1068.2004 PMC321390

[CR77] Smith KO (1964) Relationship between the envelope and the infectivity of herpes simplex virus. Proc Soc Exp Biol Med 115:814–816 14155835 10.3181/00379727-115-29045

[CR78] Starke S, Jost I, Rossbach O, Schneider T, Schreiner S, Hung L-H, Bindereif A (2015) Exon circularization requires canonical splice signals. Cell Rep 10:103–111 25543144 10.1016/j.celrep.2014.12.002

[CR79] Szabo L, Salzman J (2016) Detecting circular RNAs: bioinformatic and experimental challenges. Nat Rev Gen 17:679–692 10.1038/nrg.2016.114 PMC5565156 27739534

[CR80] Tagawa T, Gao S, Koparde VN, Gonzalez M, Spouge JL, Serquiña AP, Lurain K, Ramaswami R, Uldrick TS, Yarchoan R, Ziegelbauer JM (2018) Discovery of Kaposi sarcoma herpesvirus-encoded circular RNAs and a human antiviral circular RNA. PNAS 115:12805–12810 30455306 10.1073/pnas.1816183115 PMC6294913

[CR81] Tagawa T, Oh D, Dremel SE, Mahesh G, Koparde VN, Duncan G, Andresson T, Ziegelbauer JM (2023) A virus-induced circular RNA maintains latent infection of Kaposi’s sarcoma herpesvirus. PNAS 120:e2212864120 36724259 10.1073/pnas.2212864120 PMC9963958

[CR82] Tagawa T et al. (2023) Gene Expression Omnibus, GSE206824.

[CR83] Toptan T, Abere B, Nalesnik MA, Swerdlow SH, Ranganathan S, Lee N, Shair KH, Moore PS, Chang Y (2018) Circular DNA tumor viruses make circular RNAs. PNAS 115:E8737–e8745 30150410 10.1073/pnas.1811728115 PMC6140489

[CR84] Ungerleider N, Concha M, Lin Z, Roberts C, Wang X, Cao S, Baddoo M, Moss WN, Yu Y, Seddon M et al (2018) The Epstein Barr virus circRNAome. PLOS Pathog 14:e1007206 30080890 10.1371/journal.ppat.1007206 PMC6095625

[CR85] Wang C, Liu H (2022) Factors influencing degradation kinetics of mRNAs and half-lives of microRNAs, circRNAs, lncRNAs in blood in vitro using quantitative PCR. Sci Rep 12:7259 35508612 10.1038/s41598-022-11339-w PMC9068688

[CR86] Wang Y, Tibbetts SA, Krug LT (2021) Conquering the host: determinants of pathogenesis learned from murine gammaherpesvirus 68. Annu Rev Virol 8:349–371 34586873 10.1146/annurev-virology-011921-082615 PMC9153731

[CR87] Wu W, Ji P, Zhao F (2020) CircAtlas: an integrated resource of one million highly accurate circular RNAs from 1070 vertebrate transcriptomes. Genome Biol 21:101 32345360 10.1186/s13059-020-02018-y PMC7187532

[CR88] Yao S, Jia X, Wang F, Sheng L, Song P, Cao Y, Shi H, Fan W, Ding X, Gao SJ, Lu C (2021a) CircRNA ARFGEF1 functions as a ceRNA to promote oncogenic KSHV-encoded viral interferon regulatory factor induction of cell invasion and angiogenesis by upregulating glutaredoxin 3. PLoS Pathog 17:e1009294 33539420 10.1371/journal.ppat.1009294 PMC7888650

[CR89] Yao W, Pan J, Liu Z, Dong Z, Liang M, Xia S, Xiao Y, Cai X, Peng T, Zhou X et al (2021b) The cellular and viral circRNAome induced by respiratory syncytial virus infection. mBio 12:e03075–03021 34872355 10.1128/mBio.03075-21 PMC8649777

[CR90] Zhang X, Xu Y, Li Y, Yuan H, Liu Z, Zhang T (2022) Prevalence and correlates of Kaposi’s sarcoma-associated herpesvirus and herpes simplex virus type 2 infections among adults: evidence from the NHANES III data. Virol J 19:5 34991626 10.1186/s12985-021-01731-9 PMC8740377

[CR91] Zhao J, Lee EE, Kim J, Yang R, Chamseddin B, Ni C, Gusho E, Xie Y, Chiang CM, Buszczak M et al (2019) Transforming activity of an oncoprotein-encoding circular RNA from human papillomavirus. Nat Commun 10:2300 31127091 10.1038/s41467-019-10246-5 PMC6534539

[CR92] Zhou TC, Li X, Chen LJ, Fan JH, Lai X, Tang Y, Zhang L, Wei J (2018) Differential expression profile of hepatic circular RNAs in chronic hepatitis B. J Viral Hepat 25:1341–1351 29888838 10.1111/jvh.12944

