# Supplementary material for: Interferon induced circRNAs escape herpesvirus host shutoff and suppress lytic infection
Source: EMBO Rep. 2024 Jan 23;25(3):1541–69. doi: 10.1038/s44319-023-00051-z (PMC10933408; doi:10.1038/s44319-023-00051-z)
Supplement: Supplementary file 1 — Appendix [file 44319_2023_51_MOESM1_ESM.pdf]

Appendix - Interferon induced circRNAs escape herpesvirus host shutoff and suppress lytic infection

Appendix Figure S1 - Overrepresentation analysis (ORA) of colinear genes containing circRNAs expressed in human herpesvirus infection models page 2

Appendix Figure S2 - In silico network predictions for circRNA-miRNA-mRNA networks page 3

Appendix Figure S3 - Transcriptional activity of genes containing circRNAs upregulated after HSV-1 infection. page 4

Appendix Figure S4 - Viral endonuclease expression in transfected 293T page 5

Appendix Figure S5 - Ingenuity pathway analysis (IPA) for upregulated genes during herpesvirus lytic infection page 6

Appendix Figure S6 - Interferon stimulated circRNAs in PBMCs page 7

Appendix Figure S7 - Validation of siRNAs targeting circRELL1 page 7

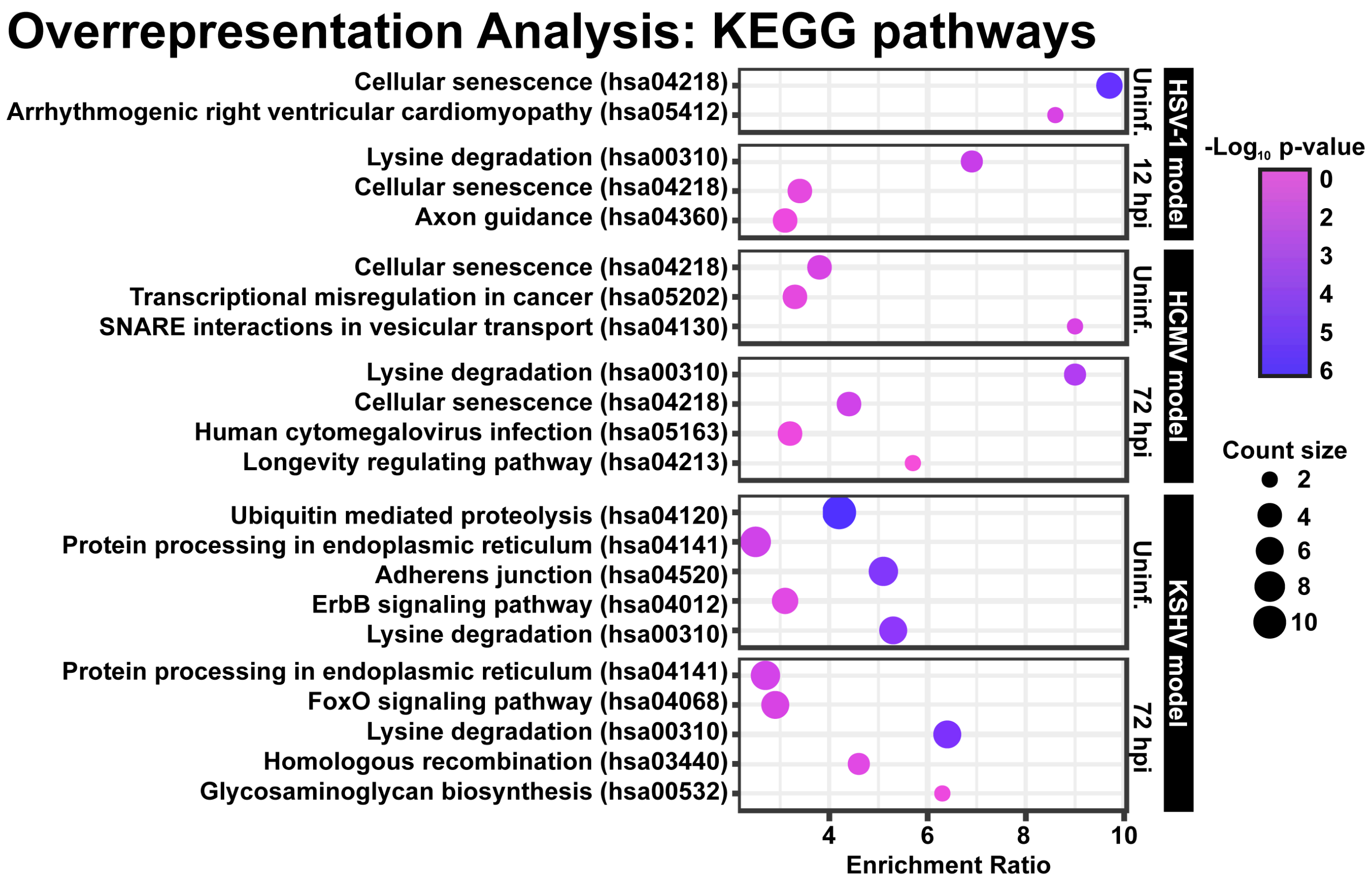

Appendix Figure S1. Overrepresentation analysis (ORA) of colinear genes containing circRNAs expressed in human herpesvirus infection models. ORA was performed using a list of genes colinear to circRNAs detected in a given model (>10 raw BSJ read counts). Data is represented as a pathway enrichment bubble plot, with bubble size indicating the number of genes from a given pathway, y-axis is enrichment ratio (relative to expected from a random gene sampling), and bubble color is -Log_{10} p-value. Only pathways with a p-value of <0.05 were included.

A

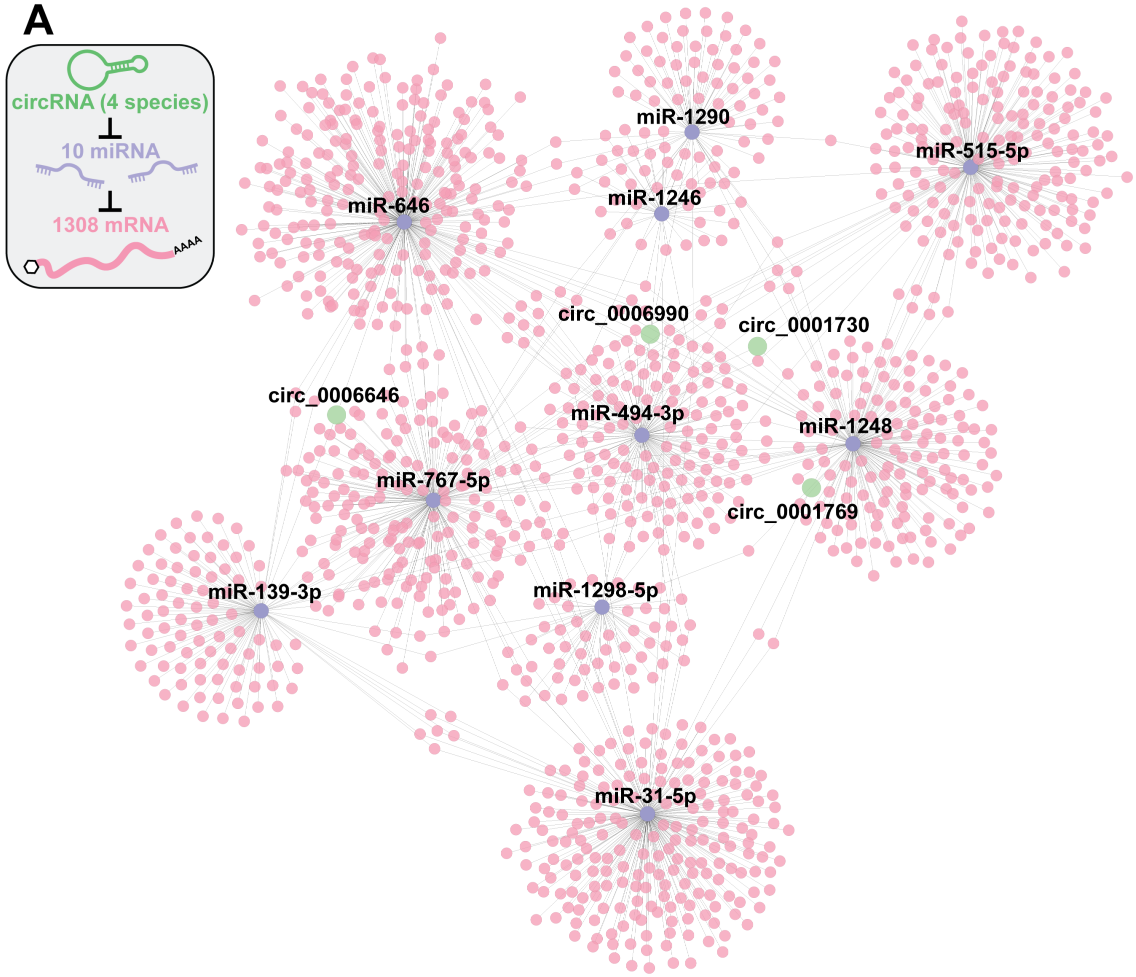

B miEAA-miRNA Enrichment Annotation

MHC class I protein complex assembly (GO0002397), p-value=2.06e-5
hsa-miR-1248; hsa-miR-515-5p; hsa-miR-1246; hsa-miR-1290; hsa-miR-1298-5p; hsa-miR-646; hsa-miR-31-5p

MHC class Ib protein complex assembly (GO0002398), p-value=2.06e-5
hsa-miR-1248; hsa-miR-515-5p; hsa-miR-1246; hsa-miR-1290; hsa-miR-1298-5p; hsa-miR-646; hsa-miR-31-5p

TAP2 binding (GO0046979), p-value=3.33e-5
hsa-miR-1248; hsa-miR-515-5p; hsa-miR-1246; hsa-miR-1290; hsa-miR-1298-5p; hsa-miR-646; hsa-miR-31-5p

TAP complex binding (GO0062061), p-value=3.35e-5
hsa-miR-1248; hsa-miR-515-5p; hsa-miR-767-5p; hsa-miR-1246; hsa-miR-1290; hsa-miR-1298-5p; hsa-miR-139-3p; hsa-miR-646; hsa-miR-31-5p

Tapasin-ERp57 complex (GO0061779), p-value=2.57e-5
hsa-miR-1248; hsa-miR-515-5p; hsa-miR-1246; hsa-miR-1290; hsa-miR-1298-5p; hsa-miR-494-3p; hsa-miR-646; hsa-miR-31-5p

Peptide antigen stabilization (GO0050823), p-value=2.06e-5
hsa-miR-1248; hsa-miR-515-5p; hsa-miR-1246; hsa-miR-1290; hsa-miR-1298-5p; hsa-miR-646; hsa-miR-31-5p

Appendix Figure S2. In silico network predictions for circRNA-miRNA-mRNA networks.
A) Four commonly-regulated human circRNAs (hsa_circ_0001730, hsa_circ_0006990, hsa_circ_0006646, hsa_circ_0001769) were examined for miRNA-binding sites using CircInteractome. Experimentally supported miRNA-mRNA interactions were then added to the network based on DIANA-TarBase v8.0. Cytoscape 3.10 was used for visualizing the network. B) Overrepresentation analysis of predicted target miRNAs was performed with miEAA2.0. Enriched Gene Ontology terms (http://geneontology.org), p-values, and miRNAs that were predicted to be regulated by circRNAs are shown.

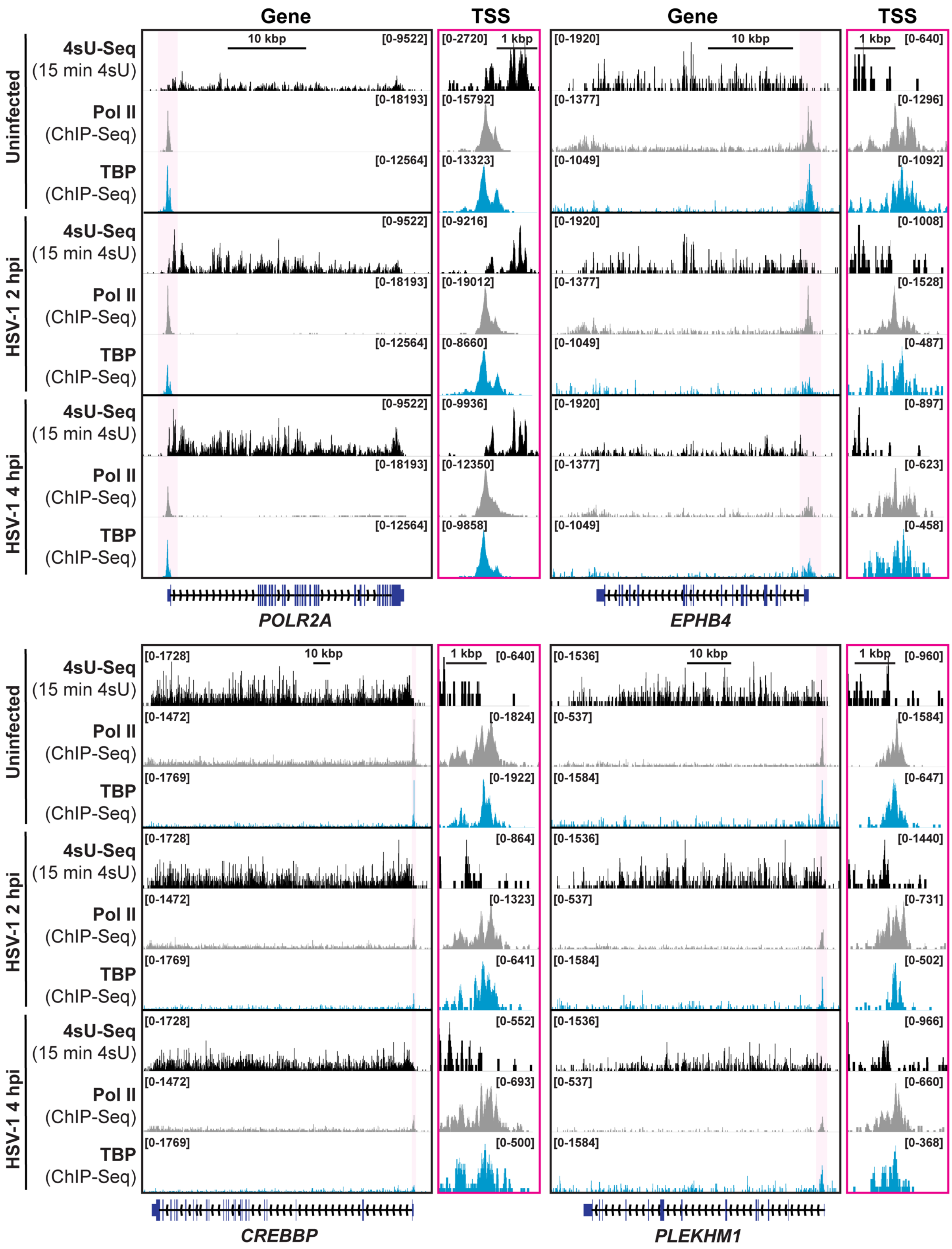

Appendix Figure S3. Transcriptional activity of genes containing circRNAs upregulated after HSV-1 infection. Nascent transcription and binding of general transcription factors for genes that host herpesvirus infection-induced circRNAs. Our previously published data from fibroblasts infected with HSV-1 (MRC-5 + KOS MOI 10) was reanalyzed (Dremel & DeLuca, 2019; Dremel *et al*, 2022). 4sU-Seq data is normalized to rRNA mapped reads. ChIP-Seq data is the average of biological duplicates and normalized to sequencing depth. Y-axes maximum and minimum values are listed within brackets. Traces spanning genes have the same y-axis maximums across infection conditions. Traces spanning 2.5 kbp transcription start sites (TSS) have autoscaled y-axis maximums.

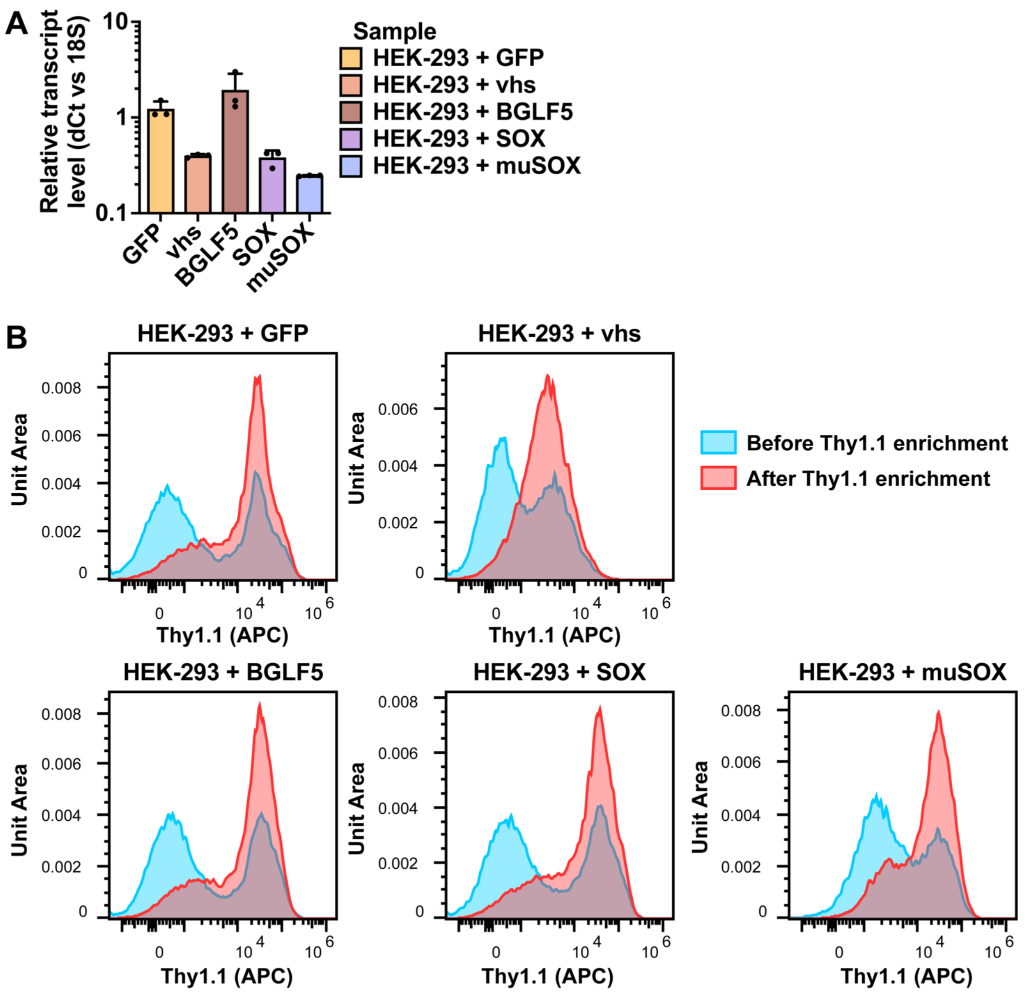

Appendix Figure S4. Viral endonuclease expression in transfected 293T.
293T cells were transfected for 24 hours with plasmid vectors expressing GFP or viral endonucleases (vhs, BGLF5, SOX, muSOX). A) RNA was collected and assessed after reverse transcription using qPCR. Data is plotted as relative transcript level (dCt) using 18S rRNA as the reference gene. Data points are biological replicates (n=3), column bars are the average, and error bars are standard deviation. B) Comparison of Thy1.1 cell surface levels assessed by flow cytometry before (blue) and after (red) magnetic sorting. Histograms of Thy1.1 signal measured using the Allophycocyanin (APC) channel, scaled to a Unit Area of 1.0 and overlaid. A representative example of three biological replicates is shown.

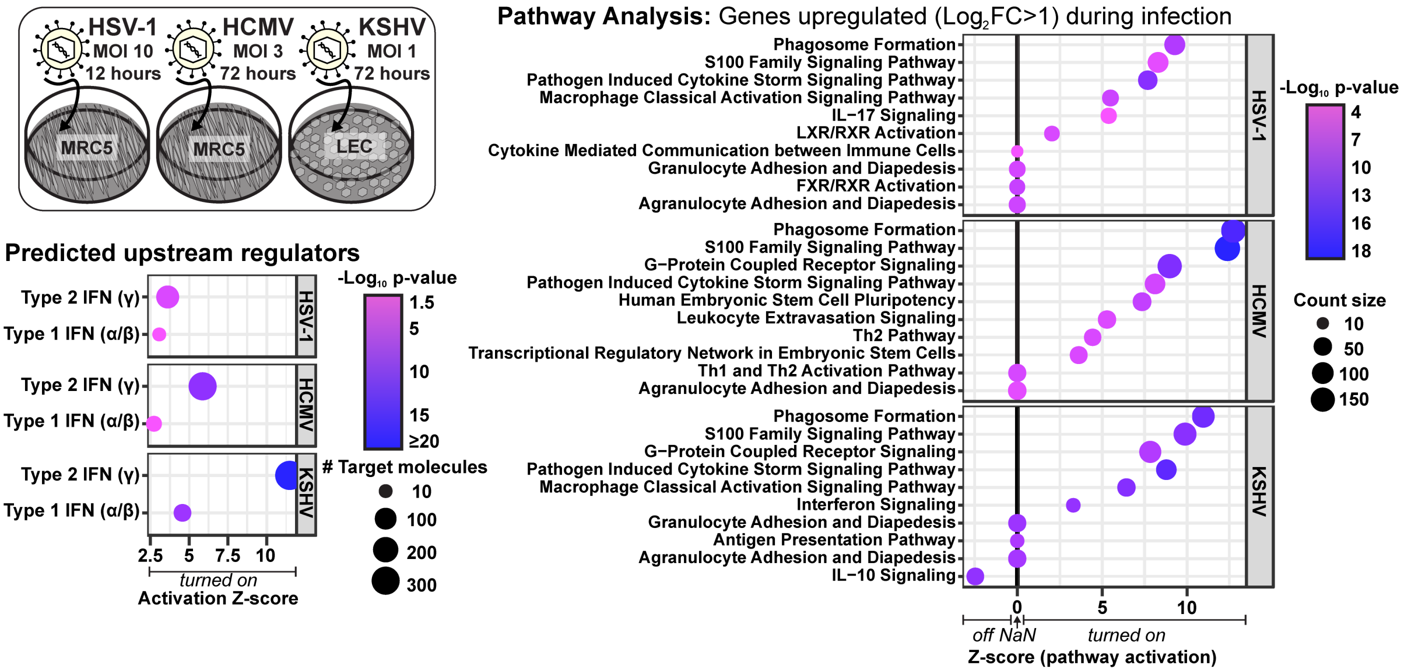

Appendix Figure S5. Ingenuity pathway analysis (IPA) for upregulated genes during herpesvirus lytic infection. IPA (Qiagen) was performed on bulk RNA-Seq datasets for HSV-1 (n=4), HCMV (n=2), and KSHV (n=2). RNA-Seq data was normalized using ERCC spike-in controls. Only genes with an average Log_2FC >1 (Infected/Uninfected) were used for IPA. The predicted upstream role of Type I and II interferon based on upregulated gene identities was presented in a bubble plot. Z-score indicates activation status, -log_{10}p-value indicates enrichment significance, and count size is the number of target molecules downstream of IFN that were upregulated. The top 10 most significantly (by p-value) altered pathways were plotted. Z-score indicates predicted pathway activation, -log_{10}p-value indicates enrichment significance, and count size is the number of upregulated genes in the pathway.

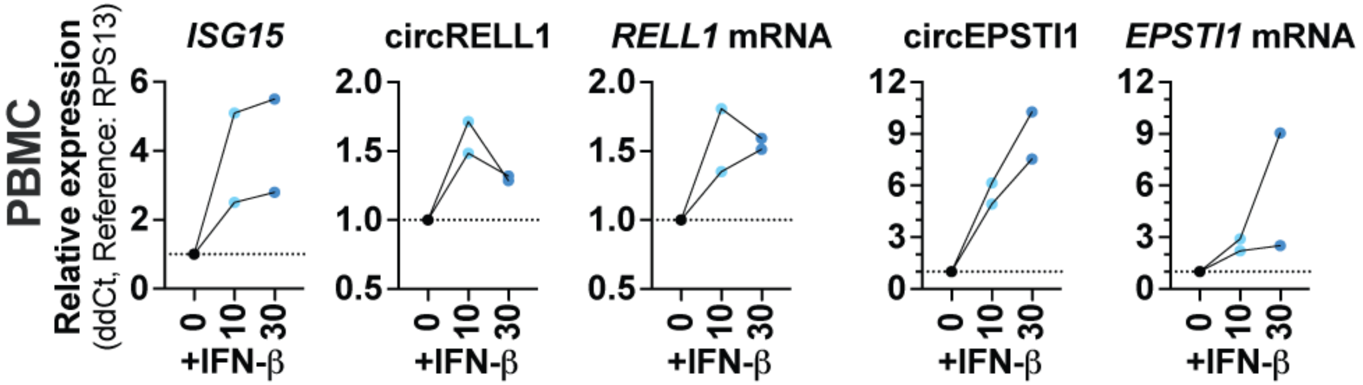

Appendix Figure S6. Interferon stimulated circRNAs in PBMCs.
Human peripheral blood peripheral blood mononuclear cells (PBMCs) were isolated from donors (n=2) and treated with IFN-β (0, 10, or 30 μg/ml) for 24 hours. RNA was collected and assessed after reverse transcription using qPCR. Data is plotted as relative expression (ddCt) using *RPS13* mRNA as the reference gene, and relative to a paired untreated sample. Each data point is a biological replicate, with data points from the same donor connected by lines.

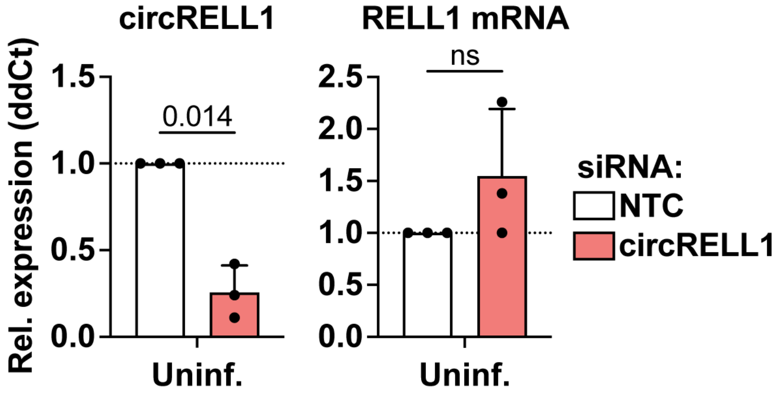

Appendix Figure S7. Validation of siRNAs targeting circRELL1.
CircRELL1 was siRNA depleted in MRC-5 cells for 48 hours. RNA was collected from the cell fraction and reverse transcribed. qPCR data is plotted as relative expression (ddCt) using 18S rRNA as the reference gene and relative to a paired Non-Targeting Control siRNA (NTC). Data points are biological replicates (n=3), column bars are the average, and error bars are standard deviation. Paired two-tailed t-tests were performed and any p-values <0.1 are listed above.
