# Supplementary material for: Interferon induced circRNAs escape herpesvirus host shutoff and suppress lytic infection
Source: EMBO Rep. 2024 Jan 23;25(3):1541–69. doi: 10.1038/s44319-023-00051-z (PMC10933408; doi:10.1038/s44319-023-00051-z)
Supplement: Supplementary file 12 — Expanded View Figures [file 44319_2023_51_MOESM12_ESM.pdf]

# Expanded View Figures

**Figure EV1. Murine circRNAs upregulated in HSV-1 infection models.**

(**A**) Infographic for murine HSV-1 infection models used in this study. (**B**) Overlap of upregulated murine circRNA detected by bulk RNA-Seq from HSV-1 latency (TG, $n = 4$), explant-induced reactivation (TG explant, $n = 3$), and drug-enhanced reactivation (TG explant + JQ1, $n = 2$). (**C**) Heatmaps for upregulated murine circRNAs, plotted as circRNA counts ($log_2FC$), Gene counts ($log_2FC$), or CIRCScore (circFPB/linearFPB). Data Information: Bulk RNA-Seq data was normalized to ERCC spike-in controls. $Log_2FC$ is relative to a paired uninfected control. Differential expression *p*-values were calculated using RankProd non-parametric permutation tests. DECs were those with raw back splice junction (BSJ) count across the sample set >10, $log_2FC > 0.5$, and $p\text{-value} < 0.05$. Heatmap values are the average of biological replicates.

© This is a U.S. Government work and not under copyright protection in the US; foreign copyright protection may apply

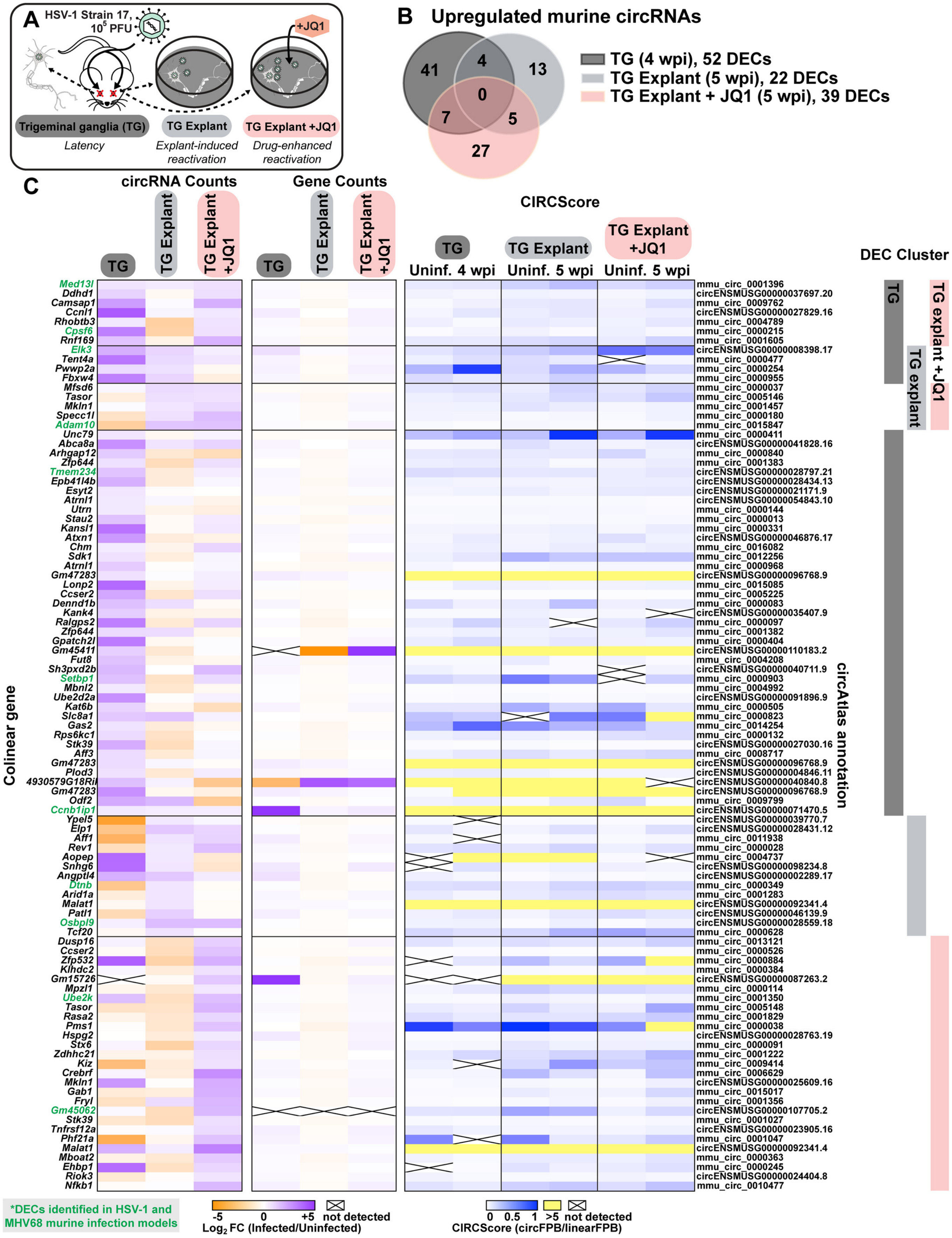


 © This is a U.S. Government work and not under copyright protection in the US; foreign copyright protection may apply

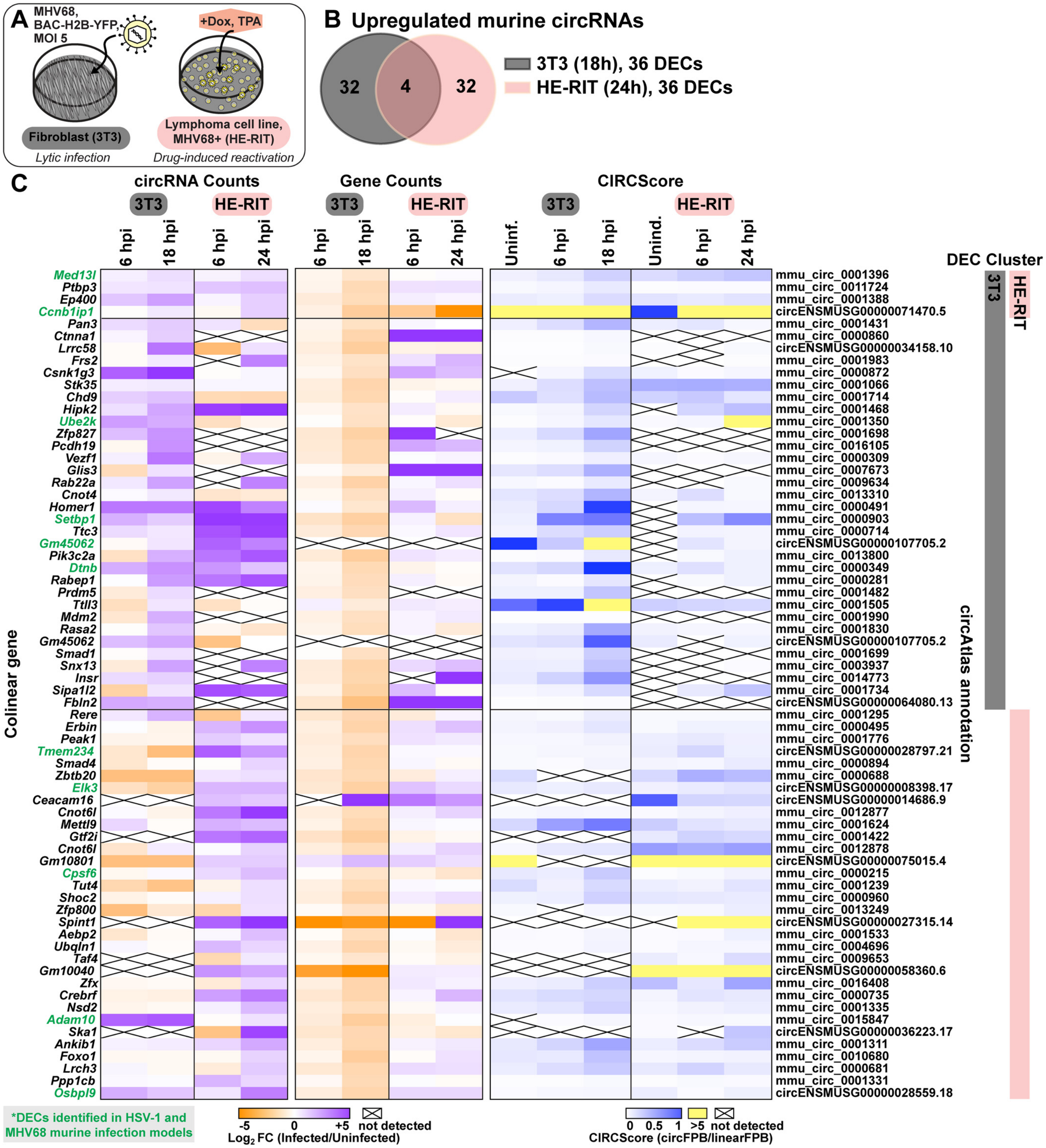


**Figure EV2. Murine circRNAs upregulated in MHV68 infection models.**

(**A**) Infographic for murine MHV68 infection models used in this study. (**B**) Overlap of upregulated murine circRNA detected by bulk RNA-Seq from MHV68 de novo infection (NIH 3T3, $n = 2$) and drug-induced reactivation (HE-RIT, $n = 2$). (**C**) Heatmaps for upregulated murine circRNAs, plotted as circRNA counts ($log_2FC$), Gene counts ($log_2FC$), or CIRCScore (circFPB/linearFPB). Bulk RNA-Seq data was normalized to ERCC spike-in controls. $Log_2FC$ is relative a paired uninfected or uninduced control. Differential expression $p$-values were calculated using RankProd non-parametric permutation tests. DECs were those with raw back splice junction (BSJ) count across the sample set >10, $log_2FC > 0.5$, and $p\text{-value} < 0.05$. Heatmap values are the average of biological replicates.

© This is a U.S. Government work and not under copyright protection in the US; foreign copyright protection may apply

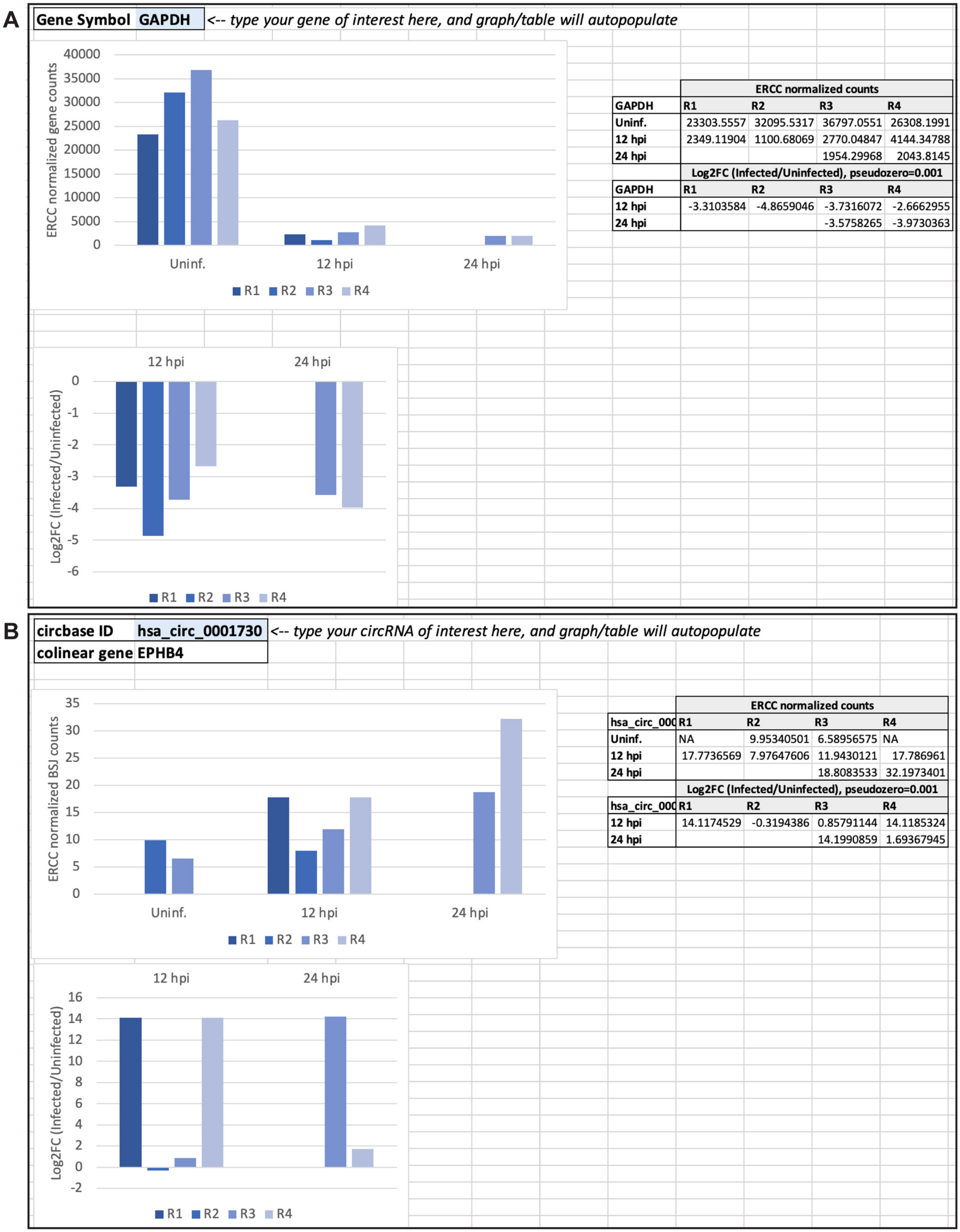


| | ERCC normalized counts | | | |
|---|---|---|---|---|
| GAPDH | R1 | R2 | R3 | R4 |
| Uninf. | 23303.5557 | 32095.5317 | 36797.0551 | 26308.1991 |
| 12 hpi | 2349.11904 | 1100.68069 | 2770.04847 | 4144.34788 |
| 24 hpi | | | 1954.29968 | 2043.8145 |
| | Log2FC (Infected/Uninfected), pseudozero=0.001 | | | |
| GAPDH | R1 | R2 | R3 | R4 |
| 12 hpi | -3.3103584 | -4.8659046 | -3.7316072 | -2.6662955 |
| 24 hpi | | | -3.5758265 | -3.9730363 |

| | ERCC normalized counts | | | |
|---|---|---|---|---|
| hsa_circ_000 | R1 | R2 | R3 | R4 |
| Uninf. | NA | 9.95340501 | 6.58956575 | NA |
| 12 hpi | 17.7736569 | 7.97647606 | 11.9430121 | 17.786961 |
| 24 hpi | | | 18.8083533 | 32.1973401 |
| | Log2FC (Infected/Uninfected), pseudozero=0.001 | | | |
| hsa_circ_000 | R1 | R2 | R3 | R4 |
| 12 hpi | 14.1174529 | -0.3194386 | 0.85791144 | 14.1185324 |
| 24 hpi | | | 14.1990859 | 1.69367945 |


© This is a U.S. Government work and not under copyright protection in the US; foreign copyright protection may apply

**Figure EV3. Screenshot of interactive resource table for transcriptomic data.**

(**A**) Representative screenshot of the GenePlotter tab where users can query any gene of interest. Supporting datasets were generated for all bulk RNA-Seq data analyzed in this study. Datasets include raw and normalized gene counts. (**B**) Representative screenshot of the CircPlotter tab where users can query any circRNA of interest. Supporting datasets were generated for all bulk RNA-Seq data analyzed in this study. Datasets include raw and normalized BSJ counts.

© This is a U.S. Government work and not under copyright protection in the US; foreign copyright protection may apply

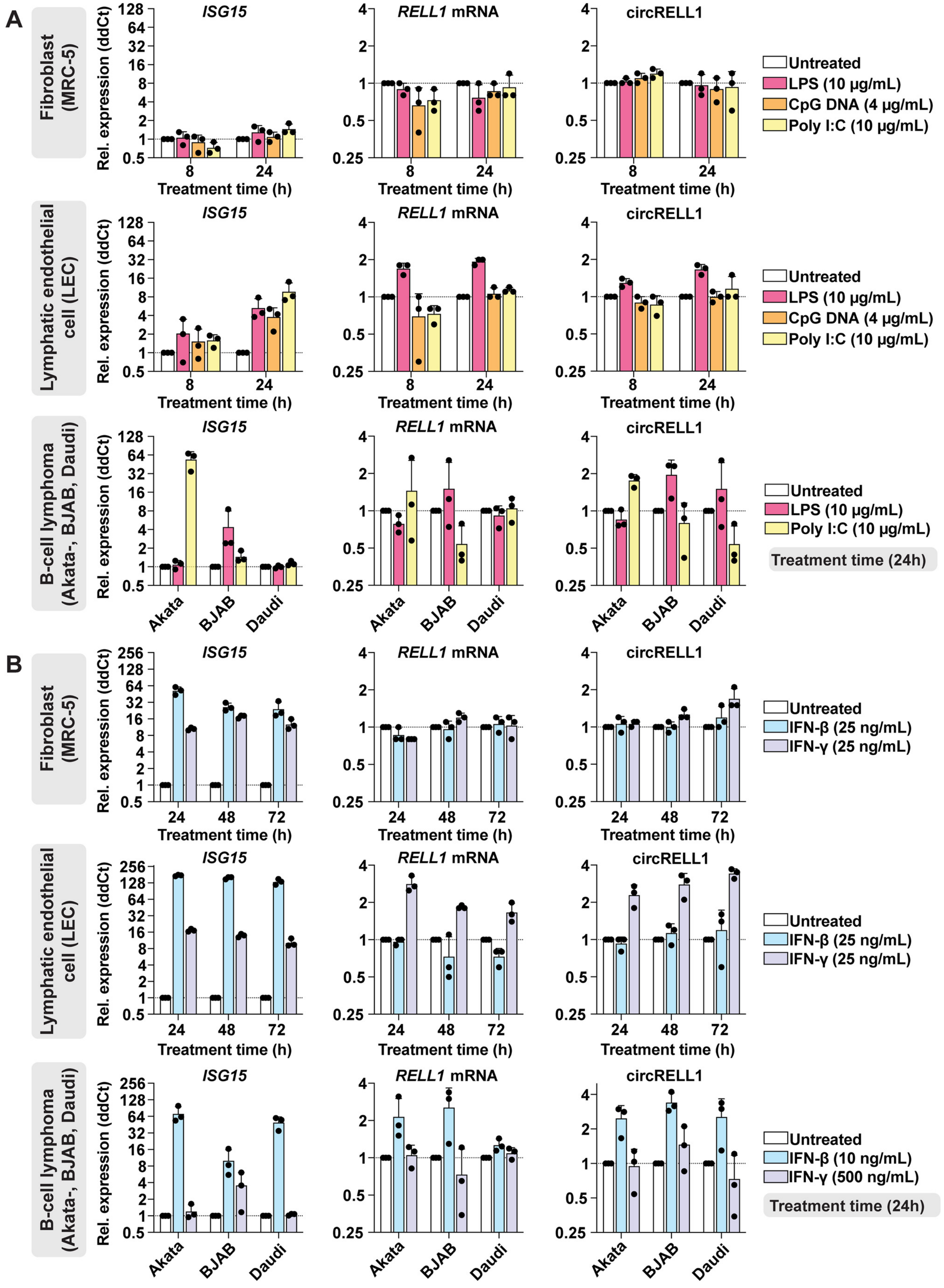



© This is a U.S. Government work and not under copyright protection in the US; foreign copyright protection may apply

**Figure EV4. CircRELL1 expression changes in response to immune stimuli.**

(**A**) Transcript quantitation for RNA collected from fibroblasts (MRC-5), lymphatic endothelial cells (LEC), or B-cell lymphoma cells (Akata-, BJAB, Daudi) treated with lipopolysaccharide (LPS), poly I:C, or CpG DNA ($n = 3$). (**B**) Transcript quantitation for RNA collected from MRC-5, LEC, or B-cell lymphoma cells treated with IFN-β and -γ ($n = 3$). Data Information: qPCR data is plotted as relative expression (ddCt) using 18S rRNA as the reference gene, and relative to a paired untreated sample. Data points are biological replicates, column bars are the average, and error bars are standard deviation.

© This is a U.S. Government work and not under copyright protection in the US; foreign copyright protection may apply

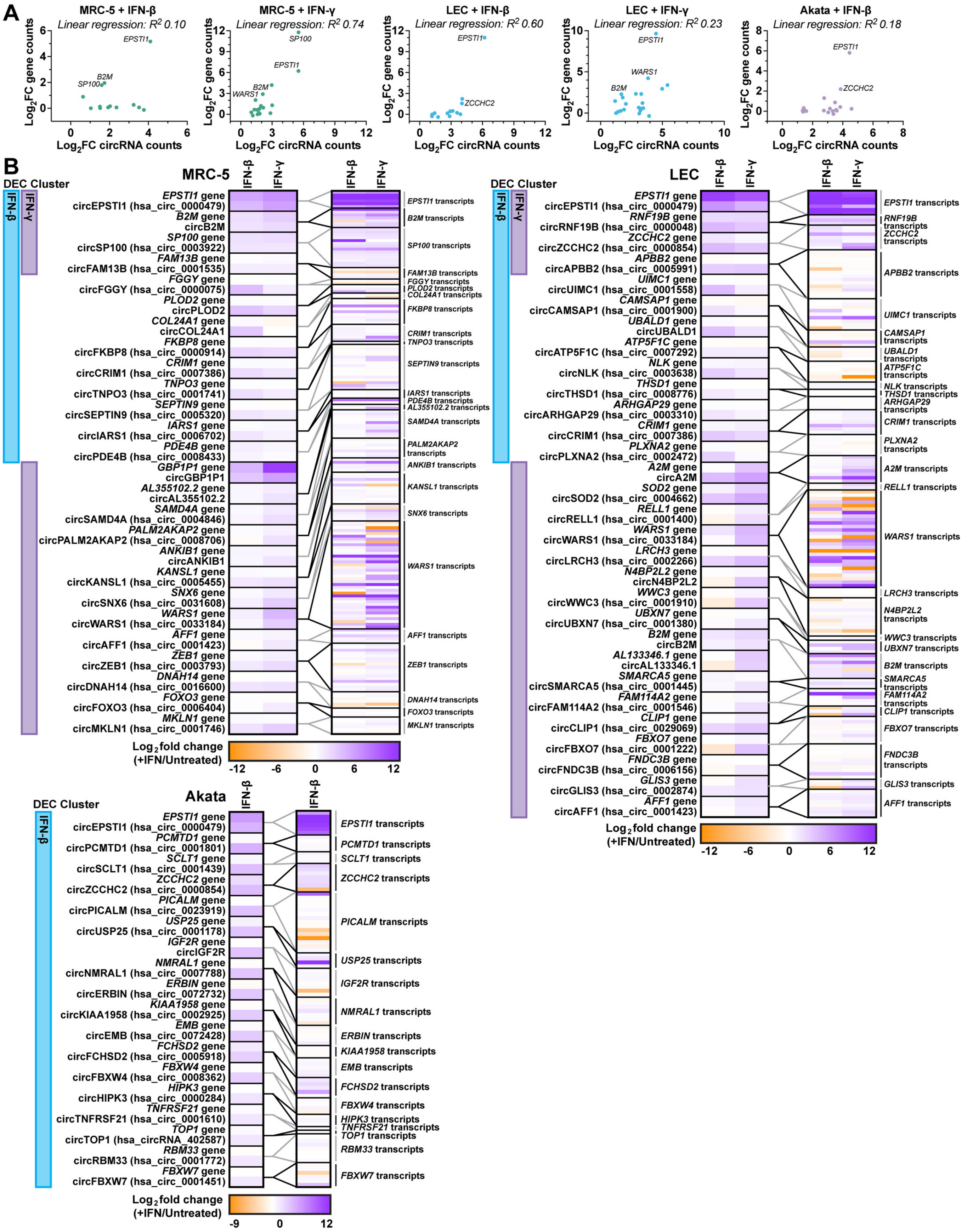


 © This is a U.S. Government work and not under copyright protection in the US; foreign copyright protection may apply

**Figure EV5. Transcript isoform analysis of interferon-stimulated cells.**

(**A**) Bulk RNA-Seq data from Fig. 4 plotting $log_2$FC (+IFN/untreated) for DECs relative to their colinear gene reads. Genes with similar levels of upregulation for circRNA and colinear gene species are labeled. (**B**) Bulk RNA-Seq data from Fig. 4 plotted as heatmaps showing $log_2$FC (+IFN/untreated) for circRNA species and their colinear gene or transcript isoforms. Gene and transcript quantitation was performed using Salmon and a reference transcriptome fasta. The sample (+IFN-β or +IFN-γ) where a differentially expressed circRNA (DEC) was identified are labeled on the left of each heatmaps as "DEC cluster". Data Information: Bulk RNA-Seq data was normalized to ERCC spike-in control and $log_2$FC was determined relative to a paired untreated sample. All data plotted is the average of biological replicates ($n = 3$). The relationship between circRNA and colinear gene expression changes was assessed by linear regression analysis, $R^2$ values are given.

© This is a U.S. Government work and not under copyright protection in the US; foreign copyright protection may apply
